# Megakaryocytic TGFβ1 orchestrates osteogenesis of LepR^+^ SSCs to alleviate radiation-induced bone loss

**DOI:** 10.1038/s12276-025-01612-z

**Published:** 2026-01-14

**Authors:** Yong Tang, Jiulin Tan, Qixiu Yu, Wenxin Yang, Zhengrong Chen, Yueqi Chen, Qiankun Yang, Jie Zhang, Qijie Dai, Bo Yu, Yunqin Xu, Linying Zhou, Gang Wang, Ce Dou, Junping Wang, Fei Luo

**Affiliations:** 1 National and Regional United Engineering Lab of Tissue Engineering, Department of Orthopaedics, Southwest Hospital, Army Medical University (Third Military Medical University), Chongqing, China; 2 Department of Orthopaedics, 72nd Group Army Hospital, Huzhou University, Huzhou, China; 3 School of Life and Health, Huzhou College, Huzhou, China; 4 State Key Laboratory of Trauma and Chemical Poisoning, Institute of Combined Injury, Chongqing Engineering Research Center for Nanomedicine, College of Preventive Medicine, Army Medical University (Third Military Medical University), Chongqing, China

**Keywords:** Mechanisms of disease, Stem-cell differentiation

## Abstract

It has been reported that a close relationship exists between the hematopoietic and skeletal systems, and megakaryocytes (MKs) may play a role in maintaining bone homeostasis. However, the precise role and underlying mechanisms of MKs in osteogenesis, particularly under stress conditions, remain largely unknown. Here we demonstrate that deficiency of MKs significantly impairs bone formation, accompanied by a reduction in the number of leptin receptor positive skeletal stem cells (LepR^+^ SSCs) in MKs conditionally deleted mice. Further investigations reveal that megakaryocytic TGFβ1 promotes the osteogenic differentiation of LepR^+^ SSCs following irradiation. Notably, thrombopoietin treatment effectively maintains the number of LepR^+^ SSCs and stimulates bone formation. Moreover, MKs-derived TGFβ1 facilitates zinc ions influx into LepR^+^ SSCs by activating Slc39a14, thereby alleviating endoplasmic reticulum stress after irradiation. In addition, the increased intracellular zinc levels inhibit PTP1B expression and activate Stat3 signaling, promoting osteogenic lineage commitment. In conclusion, our findings demonstrate that the megakaryocytic TGFβ1 orchestrates the osteogenesis of LepR^+^ SSCs following irradiation, offering a potential therapeutic strategy for radiation-induced bone loss.

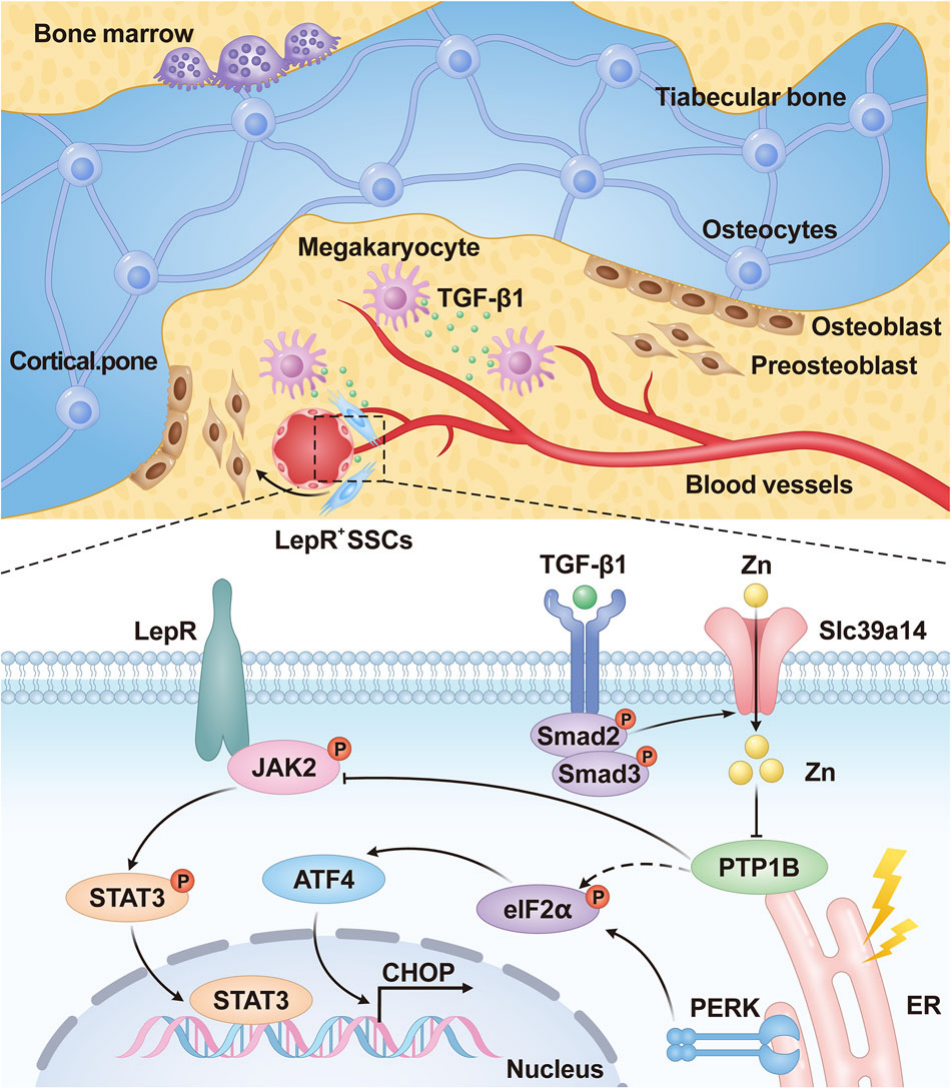

## The role of megakaryocytes in the bone microenvironment after irradiation

This study explores how certain cells in our bones, called skeletal stem cells (SSCs), help maintain bone health and repair damage. The researchers found that a small group of cells, known as leptin receptor-positive (LepR^+^) SSCs, are crucial for bone repair. The study focused on how megakaryocytes (MKs), a type of bone marrow cell, support these SSCs. The researchers used mice to study the effects of radiation on bones and how MKs help LepR^+^ SSCs recover. They discovered that MKs release a protein called TGFβ1, which helps LepR^+^ SSCs absorb zinc ions. This process reduces stress in the cells and encourages them to become osteoblasts. The study also showed that increasing MKs in the bone marrow can improve bone strength after radiation. In conclusion, MKs play a vital role in bone repair by supporting LepR^+^ SSCs. This summary was initially drafted using artificial intelligence, then revised and fact-checked by the author.

## Introduction

To maintain bone homeostasis, it is crucial to coordinate the dynamic balance between osteoblasts and osteoclasts. Skeletal stem cells (SSCs) are highly selective and homogeneous populations that can differentiate into bone and cartilage^1^. These cells are further subdivided into bone marrow (BM) SSCs, growth plate SSCs, periosteal SSCs and others^2^. SSCs not only play a key role in bone development and homeostasis but also contribute to repair of bone damage^3,4^. Although leptin receptor-positive (LepR^+^) cells constitute only 0.3% of the total BM cell population, they represent 94% of colony-forming unit fibroblasts^5^. LepR^+^ cells arise postnatally and are primarily localized around sinusoids and arterioles. As development progresses, LepR^+^ SSCs become the primary source of osteoblasts, chondrocytes and adipocytes in adulthood^6^. Lineage tracing studies have shown that LepR^+^ SSCs give rise to the majority of osteoblasts and adipocytes in the BM starting at 8 weeks of age. Although LepR^+^ SSCs contribute minimally to bone development, they serve as essential reserve cells crucial for maintaining adult bone homeostasis^6^. LepR^+^ SSCs are rapidly activated during fractures or radiation-induced damage to support the formation of bone and cartilage^5,7^. Interestingly, bone lining cells can be reactivated into cuboidal, actively bone-forming osteoblasts, expressing cell surface markers typical of SSCs, including leptin receptor^8^. However, the mechanisms by which LepR^+^ SSCs are activated in vivo remain unclear.

Skeletal damage induced by irradiation is a common side effect of radiotherapy in patients with cancer. Irradiation leads to varying degrees of bone loss and reduced bone strength^9^. However, the exact mechanisms behind radiation-induced bone loss remain incompletely understood. Irradiation causes significant changes in the bone microenvironment, including a reduction in BM cells, destruction of microvessels, production of oxidative stress and damage to intrinsic paracrine signaling factors^10^. Currently, no clinical agents have been established or translated into effective treatments for radiation-induced bone loss. Clinically, drugs that inhibit osteoclast function and promote osteoblast vitality are used, such as bisphosphonates, denosumab and teriparatide acetate^11,12^. However, none of these treatments can completely prevent radiation-induced bone loss and their long-term efficacy remains limited. As a result, novel and more effective therapeutic strategies are urgently needed.

The hematopoietic and skeletal systems support one another through a complex regulatory network^13^. However, research on the interaction between hematopoietic function and bone homeostasis remains limited. Megakaryocytes (MKs) have been shown to play a role in the reconstruction of osteogenic niches following radiation damage^14^. MKs can modulate bone metabolic balance by secreting a variety of cytokines, growth factors and other molecular regulators^15^. Previous studies have demonstrated that IGF-1 derived from MKs/platelets promotes osteogenesis, contributes to systemic IGF-1 levels, and underlies the therapeutic effects of platelet-rich plasma^16^. In addition, mice deficient in GATA-1 or NF-E2 exhibit a substantial increase in MKs, accompanied by an increase in bone trabecular number and cortical bone thickness in adulthood, although bone development itself is unaffected^17^. Surprisingly, our findings reveal that MKs contribute to bone formation by coupling osteogenesis with angiogenesis through the secretion of TGFβ1^9^. However, the mechanisms by which MKs regulate LepR^+^ SSCs in adulthood after radiation remain unclear.

In this study, we demonstrated that MKs maintain the osteogenic capacity of LepR^+^ SSCs, thereby regulating bone formation. Furthermore, our data reveal that the therapeutic effect of megakaryocytic TGFβ1 facilitates the influx of zinc ions into impaired LepR^+^ SSCs by elevating the expression of Slc39a14. This process alleviates endoplasmic reticulum (ER) stress, inhibits PTP1B expression and activates Stat3 signaling, thereby promoting the osteogenic lineage commitment of LepR^+^ SSCs after irradiation. These findings offer a new approach for treating radiation-induced bone loss.

## Materials and methods

### Animals

C57BL/6-Tg (Pf4-cre) Q3Rsko/J mice and C57BL/6-Gt (ROSA)26Sortm1(HBEGF)Awai/J (iDTR) mice were obtained from the Jackson Laboratory. Pf4-cre^+^; iDTR mice were injected with vehicle or Diphtheria toxin (DT, at the dose of 50 ng/g body weight) every 2 days. Two weeks after initial injection, these mice were used for subsequent analysis. Tgfb1tm2.1Doe/J (TGFβ1^fl/fl^) mice were purchased from Biocytogen Co. Ltd. B6.129(Cg)-Leprtm2(cre)Rck/J mice and C57BL/6JCya-Slc39a14em1flox/Cya were purchased from Cyagen Biosciences Co. Ltd. For dynamic histomorphometric analysis, mice were administered xylenol orange (90 mg/kg) 10 days and calcein (10 mg/kg) 3 days before killing. All mice were handled in accordance with the guidelines of the Institutional Animal Care and Use Committee at the Third Military Medical University (Army Medical University) (approval no. AMUWEC20210621).

### Preparation of MKs and LepR^+^ SSCs

Primary MKs and LepR^+^ SSCs were isolated according to previously published methods^18^. Primary mouse MKs were obtained by continuously enriching CD41^+^ cells and CD42d^+^ cells using an EasySep Release Mouse Biotin Positive Selection Kit and an EasySep Release Mouse PE Positive Selection Kit (all StemCell Technologies) with biotin-labeled anti-CD41 and PE-labeled anti-CD42d antibodies, according to the manufacturer’s instructions.

For primary LepR^+^ SSCs sorting, intact BM cells from femurs and tibiae were flushed and dissociated as described previously^16^. Cells were stained with an anti-LepR-biotin (R&D System, BAF497) antibody on ice for 30 min, and then the EasySep Release Mouse Biotin Positive Selection Kit (StemCell Technologies) was used according to the manufacturer’s instructions. LepR^+^ SSCs were then incubated with streptavidin-PEcy7 (Biolegend, 405206), anti-CD45-APC (eBioscience, clone: 30-F11), anti-Ter119-APC (eBioscience, clone: TER-119) and anti-CD31-APC (Biolegend, clone: MEC13.3). Cells were then centrifuged, resuspended in DAPI (Sigma-Aldrich, D9542-10MG) and sorted (DAPI^−^CD45^−^Ter119^−^CD31^−^LepR^+^) on a BD FACSAria II flow cytometer.

### Co-culture assays

For co-culture experiments, LepR^+^ SSCs were plated into 6-well plates (20 × 10^3^ cells/well). Twenty-four hours later, MKs (20 × 10^3^ cells/well) were plated into wells for direct co-culture. Specified cultures were pretreated with 100 nM of SB431542 (Sigma-Aldrich) for LepR^+^ SSCs for approximately 1 h and added at every cell media replacement simultaneously. MKs conditioned medium was obtained by removing MKs via centrifugation (5,000 rpm, 10 min). Specific experiments were treated with zinc acetate at a final concentration of 10 µM or the ER stress inhibitor 4-phenylbutyric acid (4-PBA, MCE) at a final concentration of 1 mM.

### Fluorescent indicators for zinc

The cells were incubated with the AM ester (Acetoxymethyl and acetate ester derivatives) for 60 min at 37 °C. Cells were washed in PBS (2% FBS) to remove any dye that is nonspecifically associated with the cell surface, and then incubated for a further 30 min to allow complete de-esterification of intracellular AM ester. Images were collected using a confocal laser microscope (LSM880, Carl Zeiss). To measure the total cellular zinc level, cells were sonicated to disrupt all cellular membranes, and the lysates were then incubated with the zinc fluorophore FluoZin3-AM (Invitrogen). Serum zinc was detected by a zinc content detection kit (JL-T2205).

### Establishment of the radiation-induced bone loss model

The lower limbs of mice were locally subjected to 10 Gy irradiation (γ-ray). Next, the mice were intraperitoneally treated with vehicle or thrombopoietin (TPO) (300 U/kg) every other day. Two months later, the mice were used for subsequent analyses. Pf4-cre; iDTR mice were injected daily intraperitoneally with DT and TPO for 4 weeks. The mice were injected daily intraperitoneally with the ER stress inhibitor 4-phenylbutyric acid (4-PBA, MCE) at a dose of 240 mg/kg for 4 weeks.

### Micro-CT

Micro-computed tomography (micro-CT) (Micro-CT Skyscan 1272 system, Bruker) with an isotropic voxel size of 5 µm was used to quantify the bone parameters of the femurs as previously described^9,19^. The scanning settings included a voltage of 60 kV, a current of 165 µA and a resolution of 5 µm per pixel. Reconstruction was performed using Nrecon (v1.6.10). Three-dimensional (3D) images were generated from contoured 2D images using methods based on distance transformation of the grayscale original images (CTvox, v3.0.0). The bone parameters, including trabecular bone volume fraction (BV/TV, %), trabecular number (Tb.N, 1/mm), trabecular thickness (Tb.Th, mm) and trabecular separation (Tb.Sp, mm), bone mineral density (BMD), and cortical thickness (Ct.Th, mm) were calculated using CT Analyzer (v1.15.4.0).

### Bone histomorphometry

Undecalcified femoral sections were stained with the von Kossa method or left unstained to calculate the dynamic morphometric parameters as previously described^20^. The mineral apposition rate (MAR) and bone formation rate per bone surface (BFR/BS) were analyzed with a laser confocal microscope (LSM880, Carl Zeiss). HE staining were used for the analysis of static parameters. Osteoblast surface (the percentage of TB surface covered by osteoblasts, Ob.S/BS) and osteoblast number per bone perimeter (Ob.N/B.Pm) were analyzed using ImageJ software (ImageJ, NIH).

### Biomechanics

The relative bone strength was determined by a three-point bending test with a mechanical analysis instrument (Suns) as previously described^9^. Briefly, a load was applied vertically downward at a speed of 0.05 mm/s to the middle of the femur until it broke. The peak load (N) and stiffness (N/mm) were recorded.

### Immunostaining

Immunofluorescence was performed as previously described^21^. Briefly, the bone sections were incubated with primary antibodies against mouse osteocalcin (A20800, ABclonal), Ki67 (AF7617, R&D), PTP1B (bs-55182R, Bioss), Slc39a14 (A10413, ABclonal), leptin receptor (bs-0410R, Bioss), CHOP (A21902, ABclonal), F4/80 (30325, CST), TGFβ1 (ab313729, abcam), vWF (bsm-52775R, Bioss), osterix (ab209484, abcam) and Smad2 (A7699, ABclonal) overnight at 4 °C and incubated with secondary antibodies for 1 h at 37 °C. Images were collected using a confocal laser microscope (LSM880, Carl Zeiss).

### RT–qPCR

Total RNA was isolated using TRIzol reagent (TaKaRa). Then, RNA was used to produce complementary DNA (cDNA) using the PrimeScript RT-PCR kit (A0508A) (TianGen). Next, qPCR was performed using SYBR Premix Ex Taq II (TaKaRa) according to the manufacturer’s instructions. The primer sequences are listed in Supplementary Table 1.

### scRNA-seq analysis

Single-cell RNA-sequencing (scRNA-seq) data were downloaded from the GEO dataset (GSE138689) and re-analyzed as previously reported^22^. Briefly, raw sequencing data were processed using the CellRanger pipeline (v3.0.1, 207 10X Genomics) and the expression matrix was then imported to the Seurat (v2.3.4) R toolkit for downstream analyses. After quality control and normalization, *t*-distributed stochastic neighbor embedding (*t*SNE) was used to visualize the coordinates of all cells and ‘FindAllMarkers’ was used to calculate differentially expressed genes (DEGs). The mean expression level of genes under homeostatic and stress conditions was calculated and presented by pheatmap (R package) with row-wide *z* scores. The nonlinear reconstruction algorithm DDRTree in Monocle 2 (R package) was used to infer the developmental trajectory within osteogenic lineage cells. DEGs of different osteogenic subclusters were used as the ordering genes in the pseudotime analysis.

### RNA-seq

LepR^+^ SSCs from 3 days co-cultures were collected, and RNA was isolated. RNA-seq was performed using an Illumina HiSeq2000 system. All raw data were deposited in the National Center for Biotechnology Information (NCBI) database.

### ELISAs

The expression levels of growth factors in BM were detected by enzyme-linked immunosorbent assays (ELISAs) as previously described^9^. The kits used for measuring osteocalcin and type I procollagen N-terminal propeptide (PINP) were purchased from R&D Systems.

### Western blotting

LepR^+^ SSCs were lysed using ice-cold lysis buffer containing 1% protease inhibitors and phosphatase inhibitors. Proteins were fractionated by sodium dodecyl sulfate (SDS)–polyacrylamide gel electrophoresis and then transferred to polyvinylidene difluoride (PVDF) membranes. Protein expression was detected by incubating with anti-phospho-Smad2/3 (D27F4, Cell Signaling Technology), anti-phospho-eIF2α (3398s, Cell Signaling Technology), anti-eIF2α (5324s, Cell Signaling Technology), ATF4 (11815s, Cell Signaling Technology), CHOP (2895s, Cell Signaling Technology), anti-phospho-Stat3 (9145S, Cell Signaling Technology), anti-Stat3 (33218M, Bioss), PTPN1 (55182R, Bioss), Slc39a14 (A10413, ABclonal), ALP (A0514, ABclonal), osteocalcin (A20800, ABclonal) or β-actin (AF0003, Beyotime) antibodies.

### Accurate structure prediction of biomolecular interactions with AlphaFold 3

The AlphaFold 3 model was performed as previously described^23^. Structure visualizations were created in Pymol v.2.55.5. The binding capacity of smad2 to the Slc39a14 promoter region and the binding capacity between the two proteins were predicted respectively. In addition, the binding capacity of TGFβ1 and Slc39a14 was also predicted. ChimeraX was used for molecular dynamics simulation docking.

### Statistical analysis

The results of at least three independent experiments are expressed as the mean ± SD. Differences between two groups and multiple groups were compared by two-tailed Student’s *t-*test and one-way analysis of variance (ANOVA), respectively. *P* < 0.05 was considered statistically significant.

## Results

### The maintenance of LepR^+^ SSCs was closely associated with MKs in BM

**Fig. 1:**
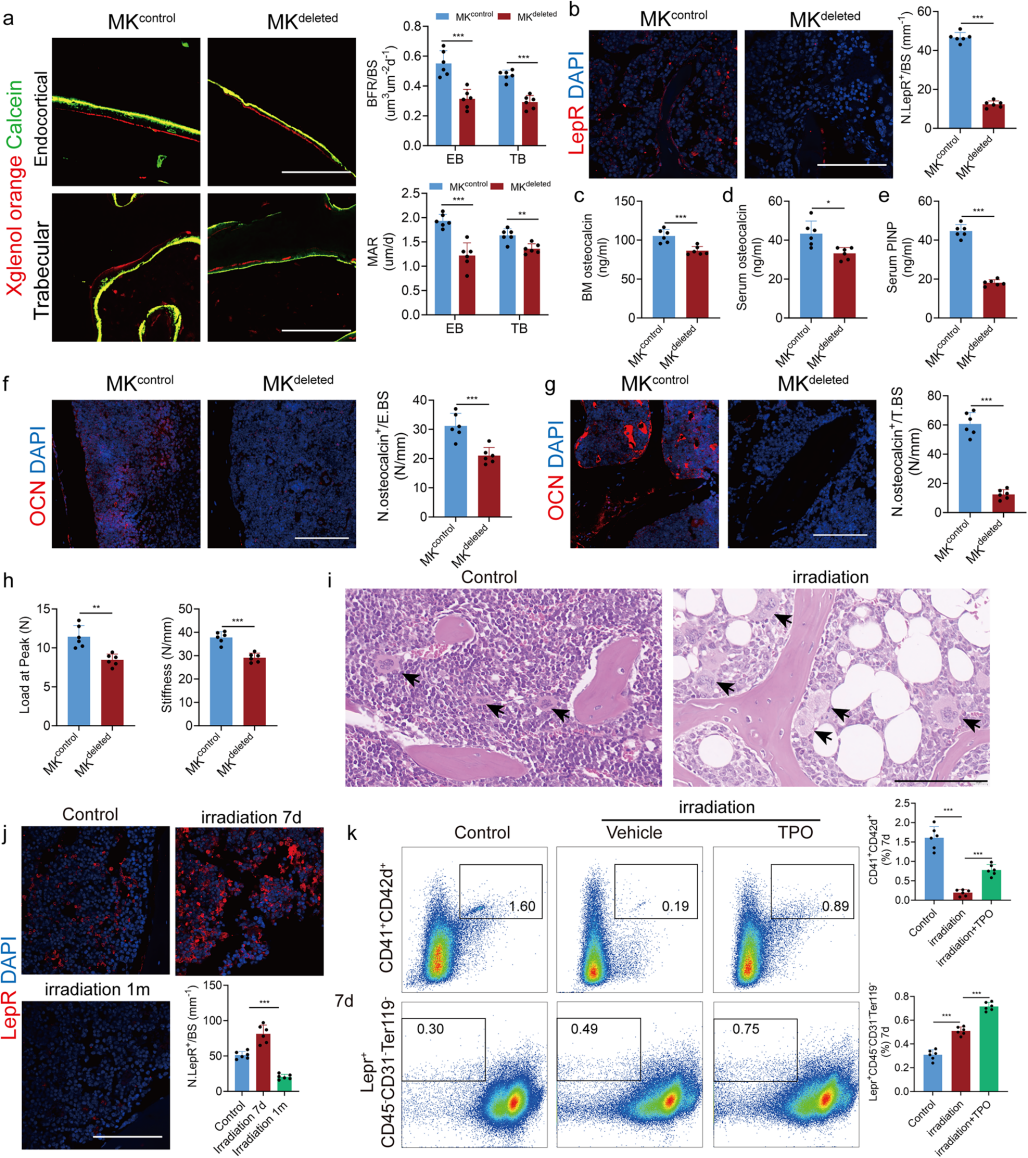
The maintenance of LepR^+^ SSCs was closely associated with MKs in BM. **a** Representative images of calcein and xylenol orange double labeling of bone and quantification of MAR and BFR for MK^deleted^ mice and their littermate controls (*n* = 6 mice per group). Scale bar, 100 µm. **b** Representative immunostaining images of LepR (red) in the BM of MK^deleted^ mice and their littermate controls. The quantification of LepR^+^ cells is shown in the right (*n* = 6 mice per group). Scale bar, 100 µm. **c** Osteocalcin concentration in the BM of MK^deleted^ mice and their littermate controls, determined by ELISA (*n* = 6 mice per group). **d** Osteocalcin concentration in the serum of MK^deleted^ mice and their littermate controls, determined by ELISA (*n* = 6 mice per group). **e** PINP in the serum of MK^deleted^ mice and their littermate controls, determined by ELISA (*n* = 6 mice per group). **f**, **g** Representative immunostaining images of OCN (red) in the EB (**f**) and TB (**g**) of MK^deleted^ mice and their littermate controls. The quantification of OCN^+^ cells is shown on the right graphs (*n* = 6 mice per group). Scale bar, 100 µm. **h** Quantitative biomechanical analysis of femora (peak load and stiffness) from MK^deleted^ mice and their littermate controls (*n* = 6 mice per group). **i** HE staining demonstrating metaphyseal bone and BM sections for MKs (black arrowheads at 48 h post-irradiation (*n* = 6 mice per group). Scale bar, 100 µm. **j** Fluorescent images of mouse femoral bone. Lepr^+^ cells (red) 7 days and 1 month after irradiation (*n* = 6 mice per group). **k** Representative flow cytometry plots and quantification of percent MKs (CD41^+^CD42d^+^) and LepR^+^ SSCs (Lepr^+^CD45^−^CD31^−^Ter119^−^) 7 days after irradiation (*n* = 6 mice per group). Data on graphs are shown as mean ± SD. An unpaired two-tailed *t*-test was used to analyze the data in **a**–**h** and one-way ANOVA was used to analyze the data in **j** and **k**. *P < 0.05, **P < 0.01 and ***P < 0.001. For all panels in this figure, data are representative of three independent experiments.

Our previous research confirmed that knockout of MKs affects the rate of bone formation, although the exact underlying mechanism remains unclear. To assess whether MKs influence LepR^+^ SSCs, we generated inducible MK-deleted mice (Pf4-cre; iDTR) by crossing Pf4-cre mice with iDTR mice (Supplementary Fig. 1a). Although a low-level of ectopic recombination of Pf4 is present, it is not significantly induced under our experimental conditions^9^. Thus, this mouse model remains a good available tool for MK-associated investigations. Histological analysis of undecalcified bone showed that both the MAR and BFR/BS were lower in MK^deleted^ mice compared to controls (Fig. 1a). Furthermore, immunofluorescence staining revealed a significant reduction in the number of LepR^+^ SSCs in the BM of MK^deleted^ mice compared to the littermate controls, with these cells primarily localized on the bone surfaces (Fig. 1b).

Next, we evaluated whether MKs affect the osteogenic microenvironment. Indeed, MKs deletion led to a decrease in osteocalcin levels in the BM (approximately 18.1%) and serum (approximately 23.7%), as well as a reduction in serum PINP (type I procollagen amino-terminal peptide) levels (approximately 59.5%) (Fig. 1c–e). In addition, the number of osteocalcin^+^ (OCN) cells on the TB (approximately 79.4%) and endocortical bone (EB) surfaces (approximately 32.7%) was reduced in MK^deleted^ mice (Fig. 1f,g). Bone histomorphometric analysis also revealed a significant decrease in Ob.S/BS (approximately 43.9%) and Ob.N/B.Pm (approximately 47.2%) in the MK^deleted^ mice compared to their littermate controls (Supplementary Fig. 1c). Furthermore, biomechanical analysis showed that bone strength and stiffness were reduced by approximately 25.9% and 22.8%, respectively, in MK^deleted^ mice (Fig. 1h).

Mature MKs were resistant to irradiation and could relocate from the central marrow space to the endosteal surface after irradiation (Fig. 1i). Flow cytometry analysis showed a 1.3-fold expansion of MKs and a 2.8-fold expansion of LepR^+^ SSCs 4 days after radiation treatment (Supplementary Fig. 1d). In the TPO treatment group, there was a 1.7-fold expansion of MKs and approximately a 7.3-fold expansion of LepR^+^ SSCs 4 days after irradiation (Supplementary Fig. 1d). After radiation damage, MKs briefly increased in the early stages, then decreased rapidly to levels lower than normal. Confocal imaging revealed dynamic changes in the LepR^+^ SSCs population after irradiation (Fig. 1j). By 7 days after irradiation, MKs had declined to 11.8% of baseline levels, accompanied by a nearly abolished expansion of LepR^+^ SSCs (only about 1.7-fold) (Fig. 1k). Although TPO treatment did not fully reverse the decline in MKs after irradiation (about 51.6%), it still significantly increased MKs compared to irradiated mice (approximately 4.1-fold) (Fig. 1k and Supplementary Fig. 1b). Interestingly, the expansion of the LepR^+^ SSCs population persisted at about 2.3-fold baseline after 7 days irradiation in mice treated with TPO (Fig. 1k). These results suggest that the maintenance of LepR^+^ SSCs is closely associated with MKs. Taken together, these data indicate that MKs and LepR^+^ SSCs may play a role in bone formation.

### TGFβ1 signaling is essential for osteogenesis in LepR^+^ SSCs of the adult skeleton after irradiation

**Fig. 2:**
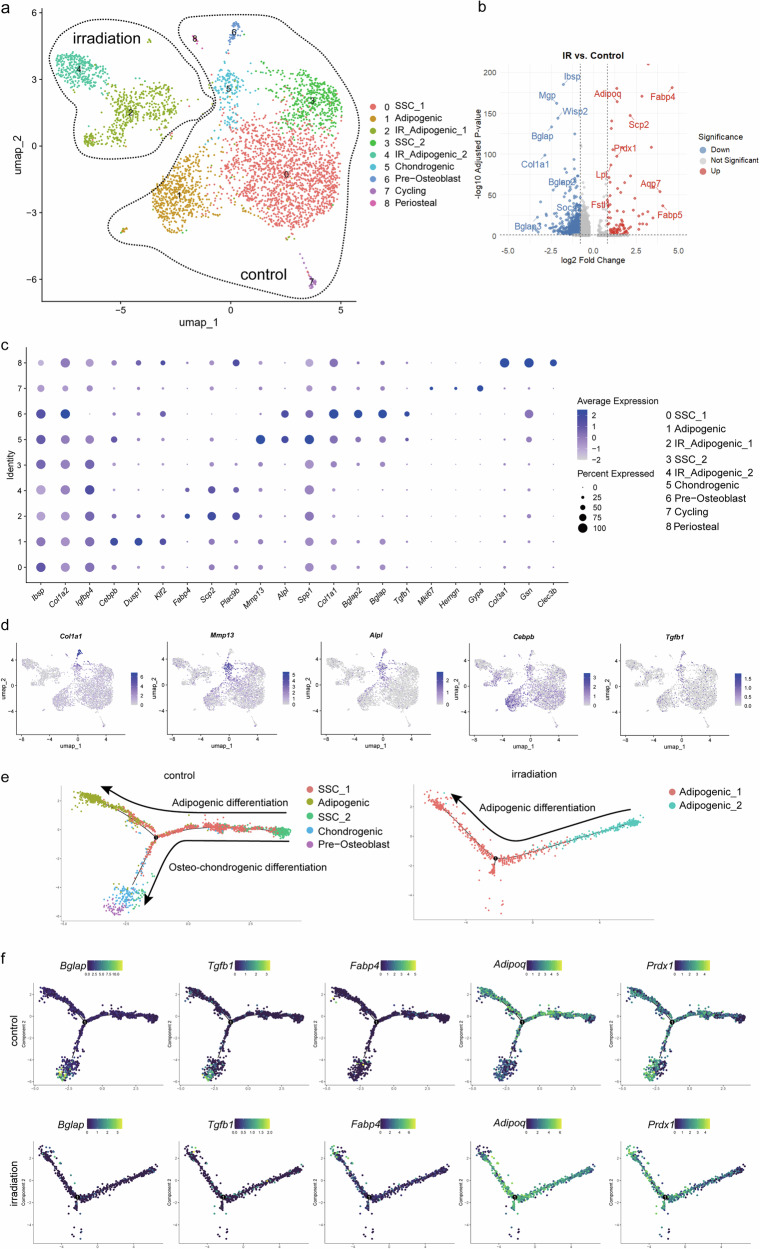
TGFβ1 signaling is essential for osteogenesis in LepR^+^ SSCs of the adult skeleton after irradiation. **a** Uniform Manifold Approximation and Projection (UMAP) plots showing CD45^−^Ter119^−^Tie2^−^Lepr^+^ single cells from control and irradiation conditions. Cells are colored by conditions and clustering. Adipogenic, osteogenic, chondrogenic, periosteal and cycling cells are highlighted by dotted lines. **b** A volcano plot displaying gene expression patterns of selected genes in the adipogenic and osteogenic clusters after irradiation (IR). **c** Dot plots displaying gene expression patterns of selected genes in the adipogenic and osteogenic clusters after irradiation. **d** Gene expression of osteogenic and adipogenic lineage-related markers under control and irradiation conditions (*Col1a1*, *Alpl*, *Mmp13*, *Cebpb* and *Tgfb1*). **e** Pseudotime analysis within Lepr-Cre-traced osteo-chondrogenic and adipogenic lineage cells under control and irradiation conditions. **f** The dynamic expression of genes related to osteo-chondrogenic and adipogenic cells in LepR^+^ SSCs along the differentiation trajectory (*Bglap, Tgfb1, Fabp4, Adipoq* and *Prdx1*).

To investigate the expression profile under irradiation conditions at single-cell resolution, we analyzed a recently generated scRNA-seq dataset of Lepr-Cre-traced cells from the long bones of adult mice. After irradiation, LepR^+^ SSCs exhibited increased adipogenesis at the expense of osteogenesis. After correcting for batch effects, we performed an integrated analysis of Lepr-Cre-traced cells under both homeostatic and irradiation conditions, revealing nine subsets (Fig. 2a and Supplementary Fig. 2a). The volcano plot showed that the expression of genes related to osteogenic differentiation were significantly downregulated and those related to adipogenic differentiation were significantly upregulated after irradiation (Fig. 2b). Following irradiation, LepR^+^ SSCs were divided into two groups, with heterogeneity nearly disappearing and the cells predominantly differentiating into adipocytes (Fig. 2a, c and Supplementary Fig. 2a). Compared to the irradiated group, the cell subsets that had disappeared tended to undergo osteogenic differentiation (Fig. 2c, d and Supplementary Fig. 2a–c). Furthermore, terminally differentiated skeletal cells, including pre-osteoblasts and chondrocytes, exhibited the highest expression of *Tgfb1* (Fig. 2c, d). Notably, the cell population with the strongest osteogenic potential disappeared, and these cells were positively expressing *Tgfb1* (Fig. 2c, d), suggesting a potential link between TGFβ1-positive cells and the osteogenic capacity of LepR^+^ SSCs. Pseudotime analysis showed that the three-lineage differentiation ability of LepR^+^ SSCs was destroyed and the ability of osteogenic and chondrogenic differentiation was impaired, but the ability of adipogenic differentiation was enhanced after irradiation (Fig. 2e). The dynamic expression of genes *Bglap* and *Tgfb1* were consistently downregulated and *Fabp4*, *Adipoq* and *Prdx1* were upregulated in LepR^+^ SSCs along the differentiation trajectory (Fig. 2f). Moreover, gene set enrichment analysis of the RNA-seq results suggested that adipogenesis, the PPARγ signaling pathway and adipogenesis activity were activated and osteoblast differentiation was downregulated after irradiation (Supplementary Fig. 2d). Taken together, these data indicated that LepR^+^ SSCs weakened their ability to differentiate into osteoblasts or chondrocytes and preferentially differentiated into adipocytes, and that TGFβ1 signaling could finetune the balance of osteogenesis and adipogenesis under homeostatic and stress conditions.

### TGFβ1 secreted by MKs promotes the osteogenic lineage commitment of LepR^+^ SSCs

**Fig. 3:**
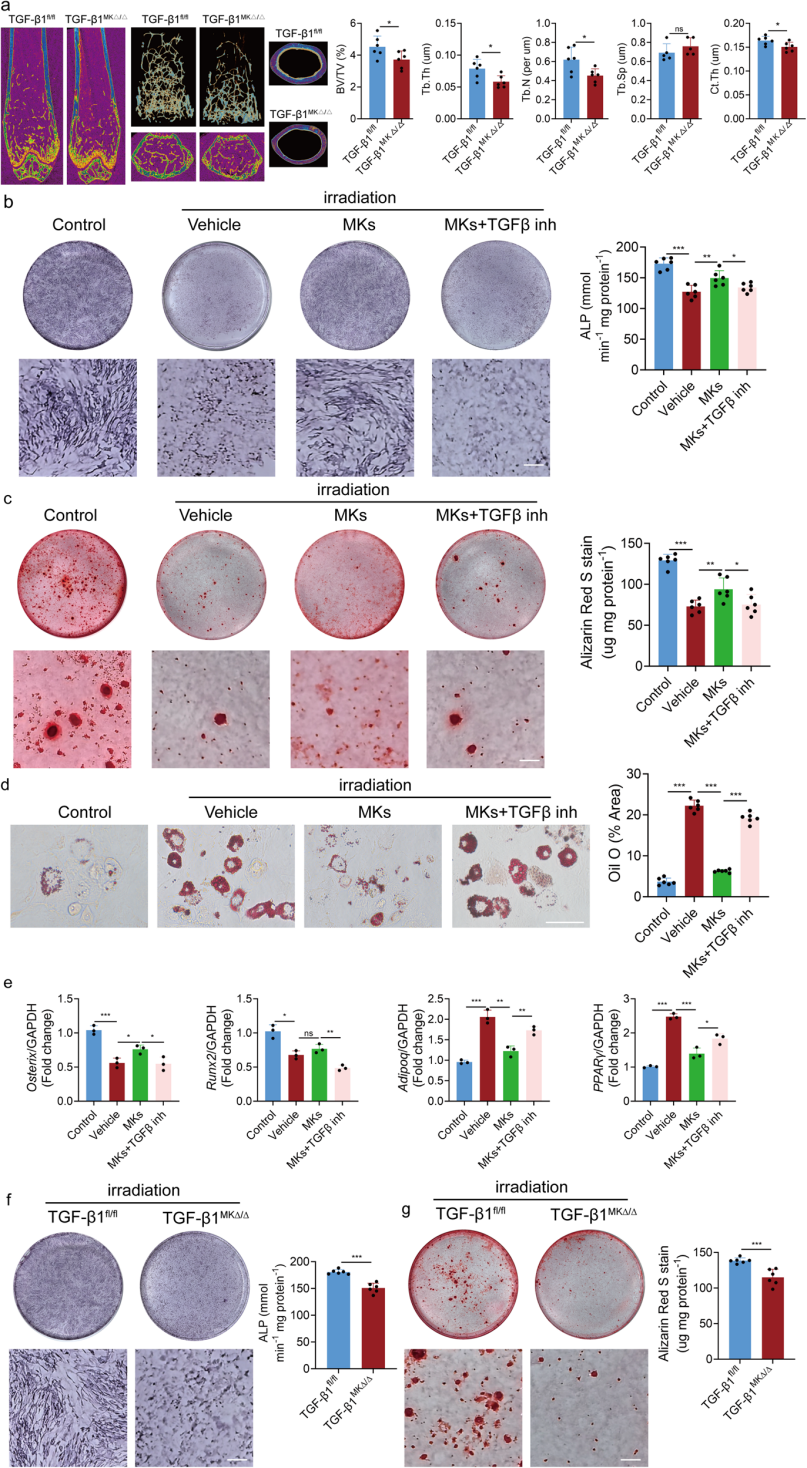
TGFβ1 secreted by MKs promotes the osteogenic lineage commitment of LepR^+^ SSCs. **a** Left: representative micro-CT images of longitudinal section femurs, cross-sectional view of the distal femurs and reconstructed trabecular structure of the region of interest from TGFβ1^MKΔ/Δ^ mice and their littermate controls (TGFβ1^fl/fl^ mice). Right: quantitative micro-CT analysis of the TB fraction (BV/TV, Tb.N, Tb.Th, Tb.Sp and Ct.Th) in TGFβ1^MKΔ/Δ^ mice and their littermate controls (TGFβ1^fl/fl^ mice) (*n* = 6 mice per group). **b** LepR^+^ SSCs were induced in osteogenic differentiation medium with or without MKs or (pretreated TGFβ type I receptor inhibitor SB431542) from wild-type (WT) mice after 14 days. Representative alkaline phosphatase staining images (left) and quantification of the activity of alkaline phosphatase was calculated (right) (*n* = 6 per group). **c** LepR^+^ SSCs were induced in osteogenic differentiation medium with or without MKs or (pretreated TGFβ type I receptor inhibitor SB431542) from WT mice after 21 days. Representative alizarin red staining images (left) and quantification of matrix mineralization was calculated (right) (*n* = 6 per group). **d** LepR^+^ SSCs were induced in adipogenic differentiation medium with or without MKs or (pretreated TGFβ type I receptor inhibitor SB431542) from WT mice after 21 days. Representative Oil O staining images (left) and the quantification of area was calculated (right) (*n* = 6 per group). **e** qPCR analysis of the expression of *Osterix*, *Runx2*, *Adipoq* and *PPARγ* in LepR^+^ SSCs with or without MKs or (pretreated TGFβ type I receptor inhibitor SB431542) from WT mice after 7 days (*n* = 3 per group). **f** LepR^+^ SSCs were induced in osteogenic differentiation medium with or without MKs from the BM of TGFβ1^MKΔ/Δ^ and TGFβ1^fl/fl^ mice after 14 days. Representative alkaline phosphatase staining images and quantification of the activity of alkaline phosphatase was calculated (*n* = 6 per group). **g** LepR^+^ SSCs were induced in osteogenic differentiation medium with or without MKs from the BM of TGFβ1^MKΔ/Δ^ and TGFβ1^fl/fl^ mice after 21 days. Representative alizarin red staining images and quantification of matrix mineralization was calculated (*n* = 6 per group). Data on graphs are shown as mean ± SD. One-way ANOVA was used to analyze the data in **a**–**e** and an unpaired two-tailed *t*-test was used to analyze the data in **f** and **g**. *P < 0.05, **P < 0.01 and ***P < 0.001. For all panels in this figure, data are representative of three independent experiments.

MKs are the primary source of TGFβ1 in the BM^24^, and our previous research demonstrated that TGFβ1 expression in MKs is higher than that of other known bone formation-related factors^9^. Our result revealed that TGFβ1 positivity was predominantly localized in MKs (Supplementary Fig. 3a, b). To further validate the role of MK-derived TGFβ1 on LepR^+^ SSCs, we specifically deleted TGFβ1 in MKs by crossing Pf4-cre mice with TGFβ1^fl/fl^ mice (hereafter referred to as TGFβ1^MKΔ/Δ^). The TGFβ1^MKΔ/Δ^ mice exhibited an overall decrease in bone remodeling, TB parameters and cortical bone thickness (Fig. 3a).

Lived nonhematopoietic and nonendothelial tdTomato^+^ cells (for Lepr-Cre) were sorted from the BM and bone fragments of 8-week-old male mice using flow cytometry. We then isolated and purified primary BM-derived MKs and found that MKs significantly induced the osteogenic differentiation of LepR^+^ SSCs and inhibited the adipogenic differentiation (Fig. 3b–e). These findings suggest that MKs-secreted factors play an important role in these processes following irradiation. To further investigate whether MK-derived TGFβ1 is involved in the osteogenic differentiation of LepR^+^ SSCs after irradiation, we employed the TGFβ type I receptor inhibitor SB431542. Notably, SB431542 significantly abolished the effect of MKs on the osteogenic differentiation of LepR^+^ SSCs (Fig. 3b–e and Supplementary Fig. 3c).

Notably, the osteogenic differentiation of the LepR^+^ SSCs were partly reduced and enhanced the adipogenic differentiation when MKs from the TGFβ1^MKΔ/Δ^ mice were added, compared to MKs from control mice, following irradiation (Fig. 3f, g and Supplementary Fig. 3d). Consistent with our previous findings, the TGFβ1^MKΔ/Δ^ mice exhibited decreased bone formation, along with reduced bone strength and stiffness^9^. Taken together, these results suggest that MK-derived TGFβ1 induces osteogenesis in mice by activating the osteogenic lineage commitment of LepR^+^ SSCs following radiation exposure.

### TGFβ1 secreted by MKs activates LepR^+^ SSCs via the Smad2/Slc39a14 signaling pathway

**Fig. 4:**
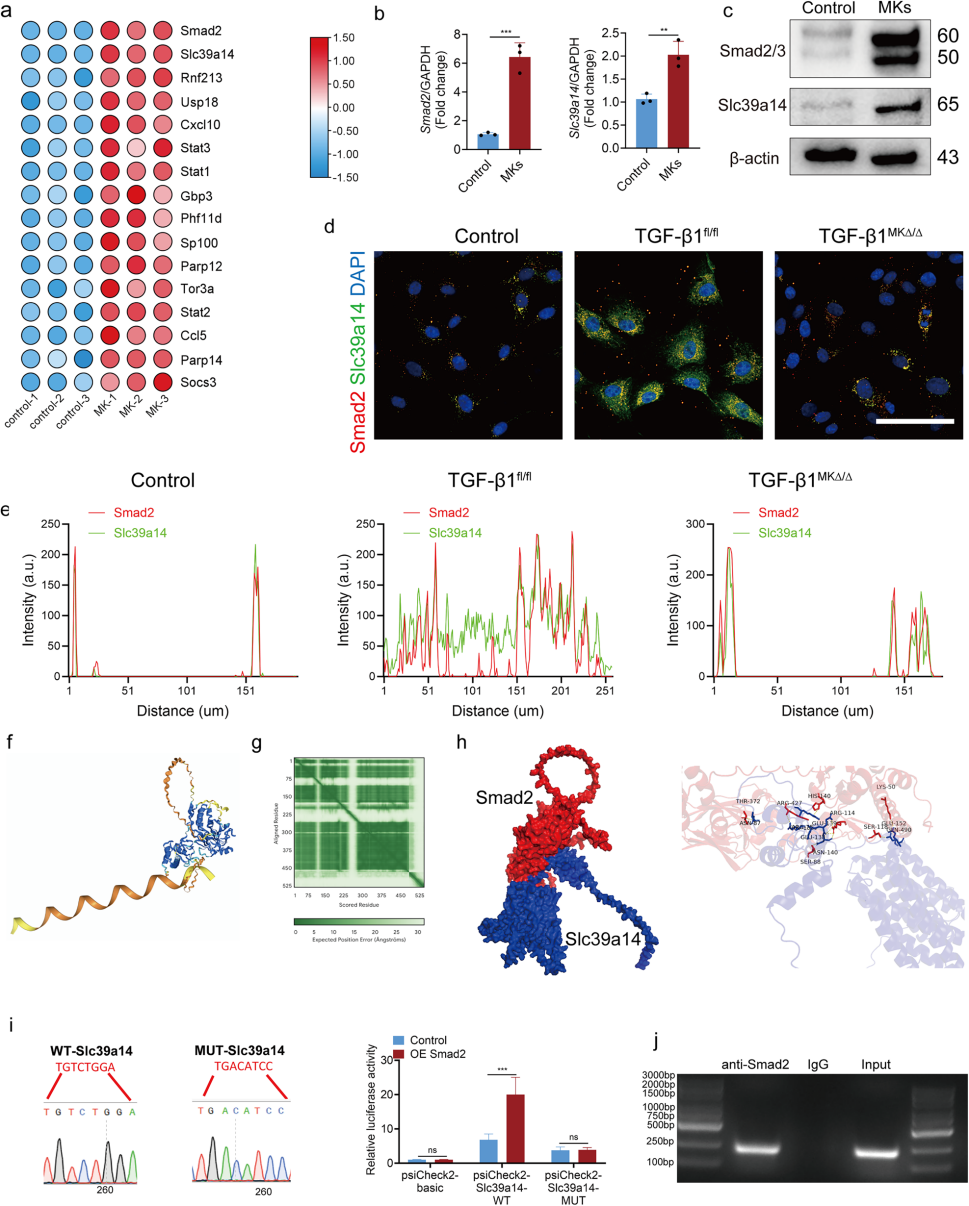
TGFβ1 secreted by MKs activates LepR^+^ SSCs via the Smad2/Slc39a14 signaling pathway. **a** RNA-seq analysis revealed changes in gene expression in LepR^+^ SSCs co-cultured with MKs (*n* = 3 each). **b** qPCR analysis of the expressions of *Smad2* and *Slc39a14* in LepR^+^ SSCs, with or without MKs, from WT mice (*n* = 6 per group). **c** Western blotting analysis of the expression of Smad2 and Slc39a14 in LepR^+^ SSCs, with or without MKs, from WT mice (*n* = 3 per group). **d** Representative immunostaining images of Smad2 (red) and Slc39a14 (green) in LepR^+^ SSCs, with or without MKs, from the BM of TGFβ1^MKΔ/Δ^ and TGFβ1^fl/fl^ mice (*n* = 6 per group). Scale bar, 100 µm. **e** Colocalization of Smad2 (red) with Slc39a14 (green) in LepR^+^ SSCs, with or without MKs, from the BM of TGFβ1^MKΔ/Δ^ and TGFβ1^fl/fl^ mice (*n* = 6 per group). Ctrl, control. **f** A schematic representation of the neural network model of Smad2 binding to the promoter region of Slc39a14, predicted by AlphaFold 3. **g** A plot of the predicted aligned error of the complex predicted by AlphaFold 3 (pTM + ipTM = 0.91). **h** A plot of the binding site and amino acid residues of Smad2–Slc39a14 analyzed by PyMol. **i** Dual-luciferase assays of 293T cotransfected with WT or mutated *Slc39a14* (LUC), combined with pcDNA3.1-Smad2 or pcDNA3.1 vetor. **j** ChIP assay of Smad2 binding to Slc39a14 promoters in LepR^+^ SSCs transfected with pcDNA3.1-Smad2 or pcDNA3.1. Immunoprecipitated DNA and the input DNA were detected by PCR. Primer sequences were designed for *Slc39a14* promoter regions located in the promoter region of the *Slc39a14* gene, with IgG as a negative control. Data on graphs are shown as mean ± SD. An unpaired two-tailed *t*-test was used to analyze the data in **b** and **i**. *P < 0.05, **P < 0.01 and ***P < 0.001. For all panels in this figure, data are representative of three independent experiments.

To investigate the mechanism by which MKs activate LepR^+^ SSCs, we performed bulk RNA-seq analysis. The results revealed that the transcription levels of *Smad2, Slc39a14, Stat1, Stat2* and *Stat3* were upregulated in LepR^+^ SSCs after coculturing with MKs (Fig. 4a). qRT–PCR and western blotting confirmed that Smad2 and Slc39a14 expression were significantly increased in LepR^+^ SSCs after co-culture with MKs (Fig. 4b, c and Supplementary Fig. 4a). Moreover, MKs from the TGFβ1^MKΔ/Δ^ mice failed to enhance the expression of Slc39a14 compared to MKs from the TGFβ1^fl/fl^ mice following irradiation (Fig. 4d, e and Supplementary Fig. 4b, c).

To explore whether Smad2 directly increases *Slc39a14* transcription, we analyzed the putative promoter region of the *Slc39a14*. Bioinformatics analysis predicted that Smad2 could bind to *Slc39a14* promoter region, as determined by AlphaFold 3 (Fig. 4f–h). Next, we introduced site-specific mutations in the Smad2 binding site within the *Slc39a1*4 promoter and confirmed that overexpression of Smad2 failed to enhance the luciferase activity of the mutated *Slc39a14*, indicating that this identified binding site is essential for Smad2 binding and the regulation of *Slc39a14* transcription (Fig. 4i). Finally, chromatin immunoprecipitation (ChIP) analysis of DNA fragments from LepR^+^ SSCs further confirmed that Smad2 directly bind to the dominant region containing the Smad2 potential binding site within the *Slc39a14* promoter (Fig. 4j). In addition, AlphaFold 3 predicted that both Smad2 and TGFβ1 could interact with the Slc39a14 protein (Supplementary Fig. 4d–m). These data reveal that TGFβ1/smad2 is involved in regulating Slc39a14 activation.

### MKs reduce ER stress in a TGFβ/Slc39a14-dependent manner

**Fig. 5:**
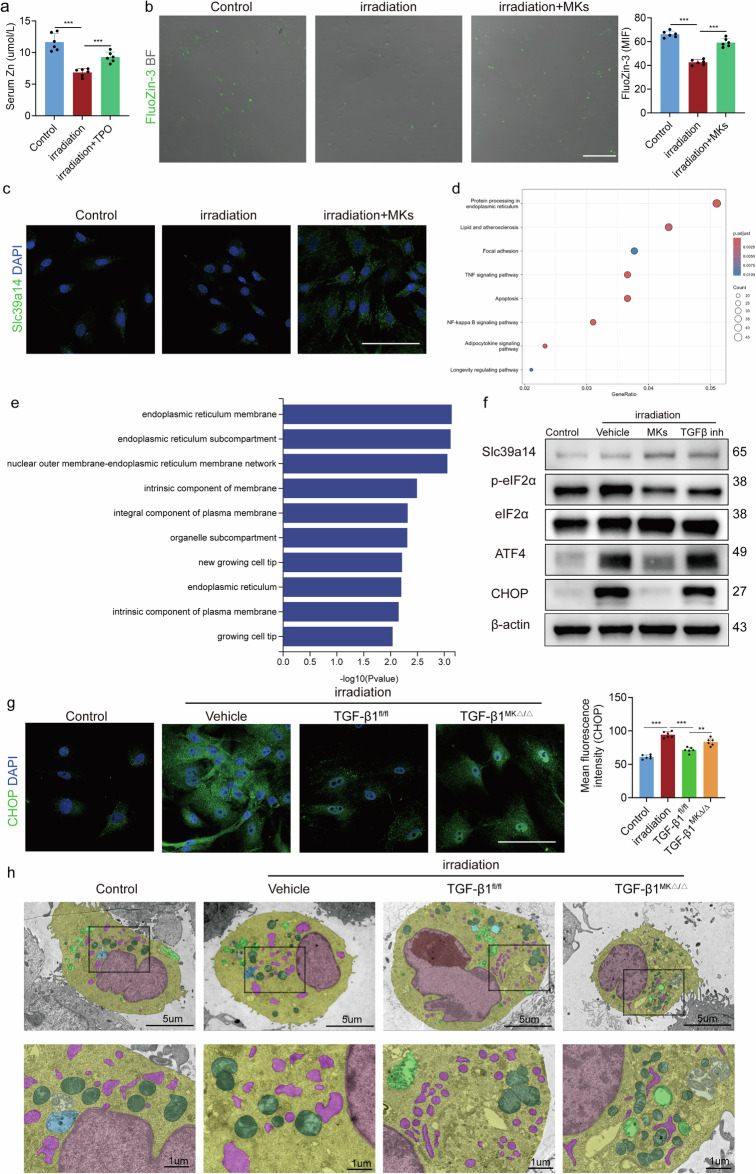
MKs reduce ER stress in a TGFβ/Slc39a14-dependent manner. **a** Serum zinc concentration in mice 4 weeks after irradiation with administration of TPO or vehicle (*n* = 6 per group). **b** Representative fluozin-3 images and quantitative analysis of LepR^+^ SSCs, with or without MKs, after irradiation (*n* = 6 per group). **c** Representative immunostaining images of Slc39a14 (green) in LepR^+^ SSCs, with or without MKs, after irradiation (*n* = 6 per group). Scale bar, 100 µm. **d** KEGG enrichment analysis of upregulated pathways in LepR^+^ SSCs after irradiation. **e** GO enrichment analysis of downregulated functions in LepR^+^ SSCs after co-culture with MKs. The top ten enriched GO terms (*P* < 0.05) are shown. **f** Western blotting analysis of the expression of Slc39a14, PTP1B, p-eIF2α, ATF4 and CHOP in LepR^+^ SSCs after co-culture with MKs (*n* = 3 per group). **g** Representative immunostaining images of CHOP (green) in LepR^+^ SSCs, with or without MKs, after irradiation (*n* = 6 per group). Scale bar, 100 µm. **h** Transmission electron microscopy images of LepR^+^ SSCs after irradiation co-culture with MKs (*n* = 3 per group). Data on graphs are shown as mean ± SD. One-way ANOVA was used to analyze the data in **a**–**d** and **g**. *P < 0.05, **P < 0.01 and ***P < 0.001. For all panels in this figure, data are representative of three independent experiments.

Slc39a14 localizes to cell membranes and promotes zinc influx into cells^25^. The zinc ion levels decreased significantly after irradiation, but this decrease could be partially reversed by treatment with TPO in vivo or co-culture with MKs in vitro (Fig. 5a, b). Although the slight increase in Slc39a14 expression after radiation damage was not statistically significant, it may represent an adaptive response to stress (Fig. 5c, f and Supplementary Fig. 5a). MKs were able to elevate Slc39a14 levels in LepR^+^ SSCs; however, in LepR^+^ SSCs that Slc39a14 specifically deleted, MKs could not increase intracellular zinc ion levels (Fig. 5c and Supplementary Fig. 5a, b).

Furthermore, Kyoto Encyclopedia of Genes and Genomes (KEGG) and Gene Ontology (GO) enrichment analysis of bulk RNA-seq revealed that functional changes related to the ER were the most prominent (Fig. 5d, e). Previous research has shown that ER stress typically occurs following irradiation^26^. The level of CHOP, a marker of ER stress, increased significantly after irradiation and was partially reduced by the addition of MKs (Fig. 5f,g). As expected, radiation damage led to elevated expression of unfolded protein response (UPR) components, including p-eIF2α, ATF4 and CHOP, indicating UPR activation (Fig. 5f). Enhanced activation of the p-eIF2α/ATF4/CHOP pathway is a hallmark of maladaptive responses to ER stress. Specifically, MKs reduced the expression of p-eIF2α, ATF4 and CHOP in LepR^+^ SSCs after irradiation through TGFβ1 (Fig. 5f,g and Supplementary Fig. 5c,d). Transmission electron microscopy confirmed that the ER was edematous and mitochondrial swelling and partial disruption of mitochondrial ridges occurred after radiation damage (Fig. 5h). Co-culturing with MKs partially alleviated ER expansion and reduced mitochondrial ridge breakage and swelling in LepR^+^ SSCs (Fig. 5h). These data demonstrate that MKs can mitigate excessive ER stress following radiation damage in LepR^+^ SSCs, alleviating both ER expansion and mitochondrial swelling.

We extended our experiments to include zinc supplementation in the absence of MKs. We found that zinc supplementation alone was sufficient to reduce ER stress levels and promote osteogenic differentiation in Slc39a14 knockout SSCs, thus recapitulating the beneficial effects observed with MK-mediated zinc transport (Supplementary Fig. 5e–g). This supports the notion that zinc’s role in alleviating ER stress and enhancing osteogenesis is independent of MKs, and is primarily driven by its capacity to modulate the cellular stress response.

### Slc39a14 inhibits the expression of PTP1B to active downstream Stat3

**Fig. 6:**
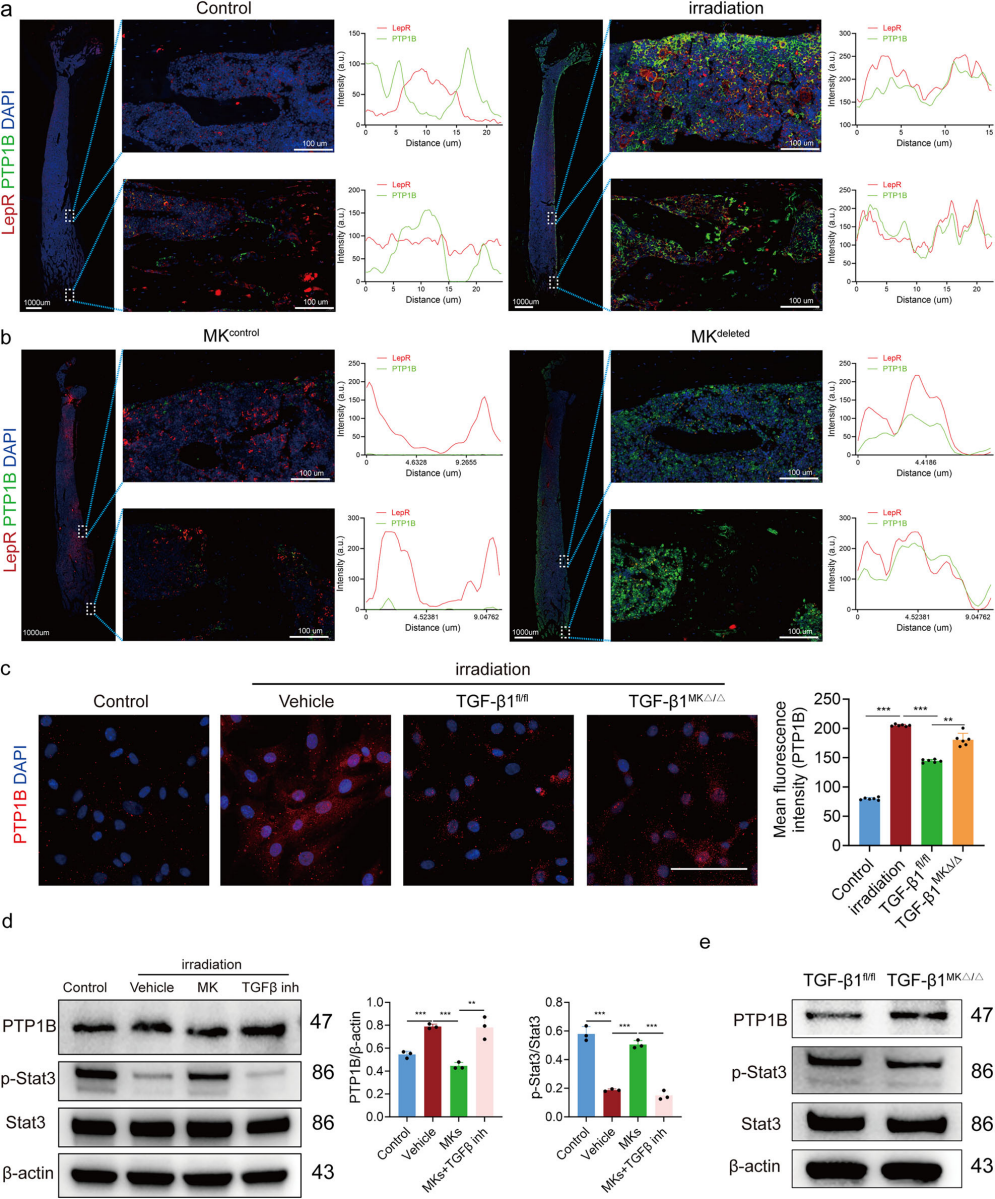
Slc39a14 inhibits the expression of PTP1B to active downstream Stat3. **a** Representative immunostaining images of LepR (red) and PTP1B (green) in the BM of irradiated mice (*n* = 6 mice per group). Scale bar, 100 µm. **b** Representative immunostaining images of LepR (red) and PTP1B (green) in the BM of MK^deleted^ mice and their littermate controls after irradiation (*n* = 6 mice per group). Scale bar, 100 µm. **c** Representative immunostaining images of PTP1B in LepR^+^ cells, with or without, MKs from the BM of TGFβ1^MKΔ/Δ^ and TGFβ1^fl/fl^ mice after irradiation (*n* = 6 per group). Scale bar, 100 µm. **d** Western blotting analysis of the expression of PTP1B and p-Stat3 in LepR^+^ SSCs after co-culture with MKs (*n* = 3 per group), inh = inhibitor. **e** Western blotting analysis of the expression of PTP1B and p-Stat3 in LepR^+^ SSCs after co-culture with MKs from the BM of TGFβ1^MKΔ/Δ^ and TGFβ1^fl/fl^ mice (*n* = 3 per group). Data on graphs are shown as mean ± SD. One-way ANOVA was used to analyze the data in **c** and **d**. *P < 0.05, **P < 0.01 and ***P < 0.001. For all panels in this figure, data are representative of three independent experiments.

PTP1B is located in the ER, and its expression increases following damage, suggesting a potential physiological role in this process^27^. Our previous study demonstrated that specific inhibition of PTP1B promotes osteogenic differentiation^28^. Consistent with these findings, we observed a significant elevation of PTP1B level in LepR^+^ SSCs after irradiation (Fig. 6a). In addition, PTP1B level was notably higher in the BM of MK^deleted^ mice than their littermate controls (Fig. 6b). Immunofluorescence and western blotting analyses indicated that MKs downregulate PTP1B expression via TGFβ1 (Fig. 6c, d). Moreover, MKs derived from TGFβ1^MKΔ/Δ^ mice showed a reduced ability to downregulate PTP1B compared to their littermate controls after radiation damage (Fig. 6e). Furthermore, the level of p-Stat3 significantly increased in LepR^+^ SSCs after co-culture with MKs (Fig. 6d, e and Supplementary Fig. 6a). Taken together, these results suggest that MK-derived TGFβ1 activates Stat3 in LepR^+^ SSCs, at least partially through the Slc39a14/PTP1B pathway, following radiation damage.

Our results confirm that inhibition of PTP1B significantly reduces Stat3 phosphorylation, whereas overexpression of PTP1B further attenuates Stat3 activation (Supplementary Fig. 6b). These findings align with previous reports suggesting that PTP1B negatively regulates Stat3 signaling^20^. Furthermore, we observed that modulating PTP1B expression, either through inhibition or overexpression, also impacts osteogenic differentiation in LepR^+^ SSCs (Supplementary Fig. 6c), consistent with the known role of Stat3 in regulating bone formation.

### MKs attenuate radiation-induced bone loss in mice by protecting LepR^+^ cells

**Fig. 7:**
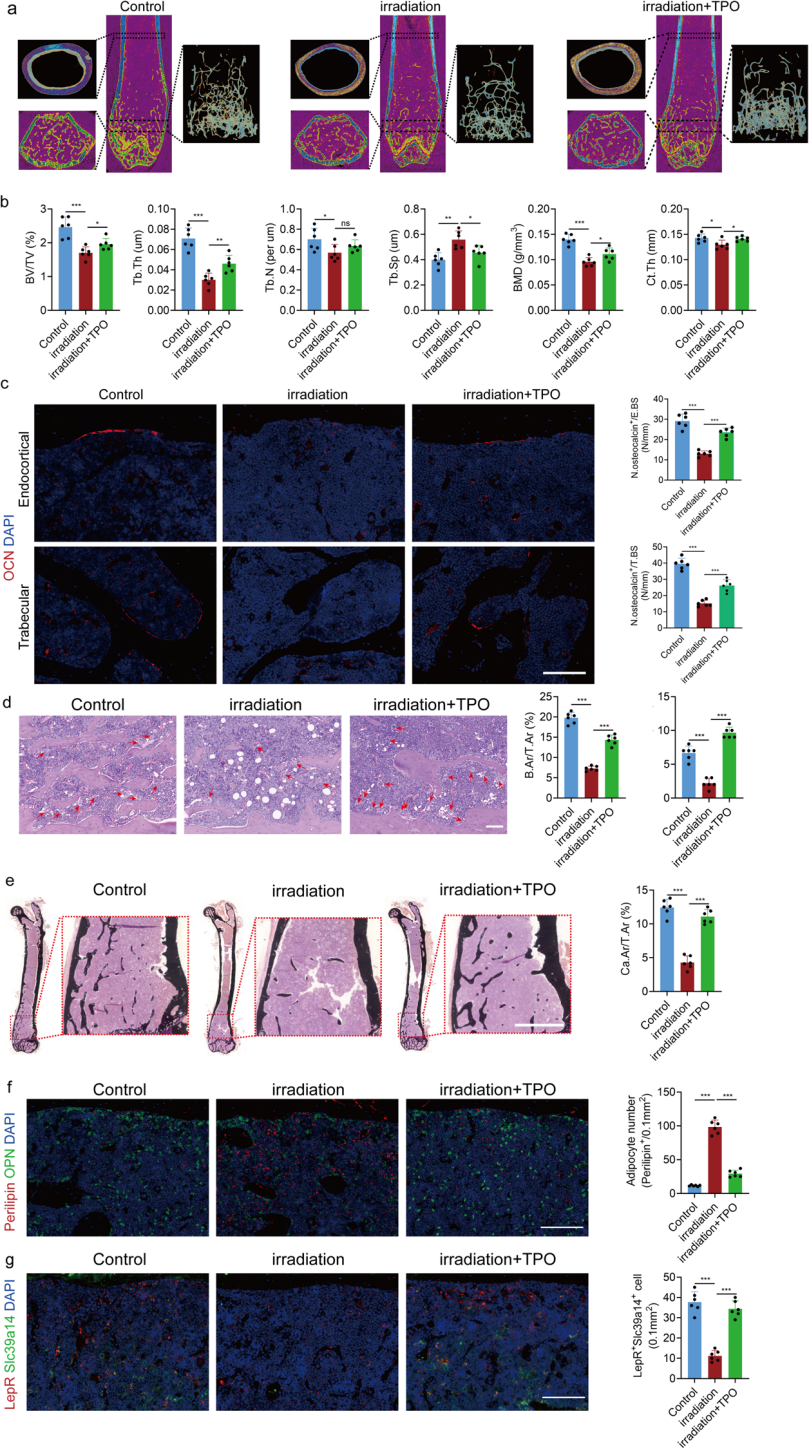
MKs attenuate radiation-induced bone loss in mice by protecting LepR^+^ cells. **a** Representative micro-CT images of longitudinal section femurs, cross-sectional view of the distal femurs and reconstructed trabecular structure of the region of interest from mice injected with TPO or vehicle after irradiation (*n* = 6 mice per group). **b** Quantitative micro-CT analysis of the TB fraction (BV/TV, Tb.N, Tb.Th, Tb.Sp, BMD and Ct.Th) in mice injected with TPO or vehicle after irradiation (*n* = 6 mice per group). **c** Representative immunostaining images of OCN (red) in the TB and EB of the mice injected with TPO or vehicle after irradiation. The quantification of OCN cells is shown on the right (*n* = 6 mice per group). Scale bar, 100 µm. **d** HE staining demonstrating B.Ar/T.Ar (bone area/total area) and the presence of MKs in the osteogenic niche of control or irradiated mice 8 weeks after injection with TPO (*n* = 6 mice per group). Scale bar, 100 µm. **e** Von Kossa staining showing mineralization of bone matrix in control or irradiated mice 8 weeks after injected with TPO (*n* = 6 mice per group). Scale bar, 1 mm. **f** Representative immunostaining images of perilipin (red) and osteopontin (OPN, green) in the BM of irradiation mice (*n* = 6 mice per group). Scale bar, 100 µm. **g** Colocalization of LepR (red) with Slc39a14 (green) in the BM of irradiation mice (*n* = 6 mice per group). Scale bar, 100 µm. Data on graphs are shown as mean ± SD. One-way ANOVA was used to analyze the data in **b**–**g**. *P < 0.05, **P < 0.01 and ***P < 0.001. For all panels in this figure, data are representative of three independent experiments.

Radiation-induced bone loss is associated with decreased bone formation. To investigate whether increasing the abundance of MKs can alleviate radiation-induced bone loss, we irradiated the limbs of mice with 10 Gy locally and treated them with intraperitoneal TPO injections to increase MK numbers in the BM (Supplementary Fig. 7a). Micro-CT analysis revealed that TPO treatment enhanced bone formation and strength in the irradiated mice (Fig. 7a,b). Immunofluorescence showed that the number of OCN^+^ cells in the BM of TPO-treated mice was maximally preserved after irradiation (Fig. 7c). Bone histomorphometric analysis further demonstrated that the irradiated mice treated with TPO had more new bone formation (Fig. 7d and Supplementary Fig. 7b). In addition, HE staining demonstrated more MKs resided in the osteogenic niche of the irradiated mice 8 weeks after injection with TPO (Fig. 7d). Notably, the irradiated mice treated with TPO exhibited a significant increase in bone mineralization (Fig. 7e), accompanied by partly retained the proliferative capacity of LepR^+^ SSCs (Supplementary Fig. 7c). To test whether MKs-derived TGFβ1 regulates BM adipogenesis under the irradiation condition, we performed immunostaining of the adipocyte maker Perilipin on femur sections (Fig. 7f). The number of BM adipocytes was significantly decreased in the irradiated mice with TPO treatment (Fig. 7f). Surprisingly, the number of osteoclasts in the femur was also reduced in the TPO-treated group (Supplementary Fig. 7d). TUNEL staining showed that systemic TPO delivery protected LepR^+^ cells from radiation-induced apoptosis (Supplementary Fig. 7e). Furthermore, the expression of Slc39a14 decreased after irradiation but could be partially restored by TPO injection (Fig. 7g). Moreover, we employed Pf4-cre; iDTR mice and administered diphtheria toxin to specifically ablate MKs, followed by injection of an equivalent dose of TPO. Our results demonstrated that bone mass was not restored (Supplementary Fig. 7f,g), suggesting that the regulatory effect of TPO on bone formation is predominantly mediated through MKs. These findings suggest that the systemic administration of TPO, which increases MKs, enhances osteogenic potential and partially inhibits osteoclastic activity.

### MKs induce osteogenic lineage commitment of LepR^+^ SSCs via Slc39a14 after irradiation

**Fig. 8:**
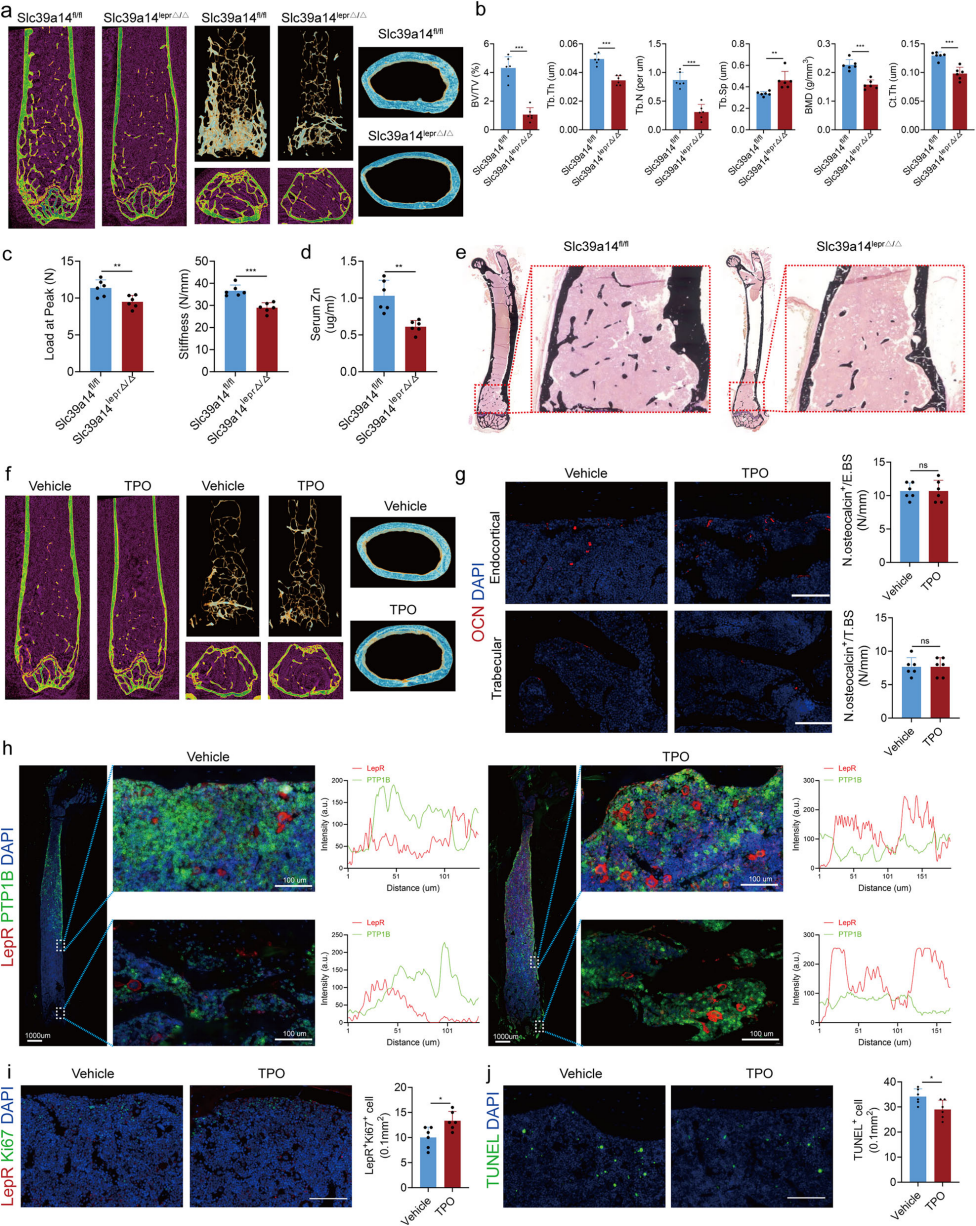
MKs induce osteogenic lineage commitment of LepR^+^ SSCs via Slc39a14 after irradiation. **a** Representative micro-CT images of longitudinal section femurs, cross-sectional view of the distal femurs and reconstructed trabecular structure of the region of interest from Slc39a14^leprΔ/Δ^ mice and their littermate controls (Slc39a14^fl/fl^ mice) (*n* = 6 mice per group). **b** Quantitative micro-CT analysis of the TB fraction (BV/TV, Tb.N, Tb.Th, Tb.Sp, BMD and Ct.Th) in Slc39a14^leprΔ/Δ^ mice and their littermate controls (Slc39a14^fl/fl^ mice) (*n* = 6 mice per group). **c** Quantitative biomechanical analysis of femora (peak load and stiffness) from MK^deleted^ mice and their littermate controls (Slc39a14^fl/fl^ mice) (*n* = 6 mice per group). **d** Serum zinc concentration in Slc39a14^leprΔ/Δ^ mice and their littermate controls (Slc39a14^fl/fl^ mice) (*n* = 6 per group). **e** Von Kossa staining showing mineralization of bone matrix in Slc39a14^leprΔ/Δ^ mice and their littermate controls (Slc39a14^fl/fl^ mice) (*n* = 6 per group). **f** Representative micro-CT images of longitudinal section femurs, cross-sectional view of the distal femurs and reconstructed trabecular structure of the region of interest from Slc39a14^leprΔ/Δ^ mice injected with TPO or vehicle after irradiation (*n* = 6 mice per group). **g** Representative immunostaining images of OCN (red) in the TB and EB of the Slc39a14^leprΔ/Δ^ mice injected with TPO or vehicle after irradiation. The quantification of OCN cells is shown on the right (*n* = 6 mice per group). Scale bar, 100 µm. **h** Representative immunostaining images of LepR (red) and PTP1B (green) in the BM of the Slc39a14^leprΔ/Δ^ mice injected with TPO or vehicle after irradiation. Scale bar, 100 µm. **i** Colocalization of LepR (red) with Ki67 (green) in the BM of the Slc39a14^leprΔ/Δ^ mice injected with TPO or vehicle after irradiation (*n* = 6 mice per group). Scale bar, 100 µm. **j** Representative immunostaining images of TUNEL (green) in the BM of the Slc39a14^leprΔ/Δ^ mice injected with TPO or vehicle after irradiation. The quantification of tunel positive cells is shown on the right (*n* = 6 mice per group). Scale bar, 100 µm. Data on graphs are shown as mean ± SD. An unpaired two-tailed *t*-test was used to analyze the data in **b**–**d**, **g**, **i** and **j**. *P < 0.05, **P < 0.01 and ***P < 0.001. For all panels in this figure, data are representative of three independent experiments.

To further investigate the role of Slc39a14 on LepR^+^ SSCs, we specifically deleted Slc39a14 in LepR^+^ cells by crossing Lepr-cre mice with Slc39a14^fl/fl^ mice (hereafter referred to as Slc39a14^leprΔ/Δ^). The Slc39a14^leprΔ/Δ^ mice exhibited decreased bone formation, as well as reduced bone strength and stiffness (Fig. 8a–c and Supplementary Fig. 8a). The zinc ion level was reduced by approximately 40.8% in Slc39a14^leprΔ/Δ^ mice (Fig. 8d). Von Kossa staining showed that Slc39a14^leprΔ/Δ^ mice had lower bone mineralization compared to their littermate controls (Fig. 8e).

To confirm that MKs regulate LepR^+^ SSCs through Slc39a14, we irradiated Slc39a14^leprΔ/Δ^ mice and injected them with TPO intraperitoneally. Micro-CT analysis showed no significant improvement in TB volume fraction, trabecular number or trabecular spacing, except the thickness of cortical bones in the mid-diaphysis (Fig. 8f and Supplementary Fig. 8b), and there were no changes in bone strength and stiffness (Supplementary Fig. 8c). The expression of PTP1B was not significantly downregulated and the number of OCN^+^ cells did not increase significantly in the slc39a14^leprΔ/Δ^ mice treated with TPO after irradiation (Fig. 8g,h). The number of BM adipocytes was no change in the Slc39a14^leprΔ/Δ^ mice with TPO treatment following irradiation (Supplementary Fig. 8d). Bone histomorphologic analysis revealed no substantial enhancement in new bone formation (Supplementary Fig. 8e,f). However, the number of LepR^+^ SSCs slightly increased in the Slc39a14^leprΔ/Δ^ mice injected with TPO following irradiation (Fig. 8h,i). TUNEL staining indicated that systemic TPO delivery protected LepR^+^ SSCs from radiation-induced apoptosis in the Slc39a14^leprΔ/Δ^ mice (Fig. 8j). Importantly, MKs were unable to reduce excessive ER stress or activate the Stat3 signaling pathway in LepR^+^ SSCs when Slc39a14 was deleted (Supplementary Fig. 8g).

To further verify whether ER stress is a key factor contributing to the impaired osteogenic capacity of LepR^+^ SSCs after irradiation, we administered the ER stress inhibitor 4-phenylbutyric acid (4-PBA) to irradiated LepR^+^ SSCs. Through alizarin red and immunofluorescence staining, we observed that 4-PBA significantly improved the osteogenic differentiation ability of LepR^+^ SSCs in vitro (Supplementary Fig. 8h,i). Furthermore, rescue experiments were performed by administering 4-PBA to irradiated mice for 4 weeks. Micro-CT results showed that the bone mass of the 4-PBA-treated group was significantly higher than the sham group (Supplementary Fig. 8j,k). These findings align with the existing literature, suggesting that inhibition of ER stress can enhance osteogenic lineage commitment in BM stromal cells^29^. Collectively, our results demonstrate that MKs induce the osteogenic lineage commitment of LepR^+^ SSCs via Slc39a14, which increases zinc ions and attenuates ER stress after irradiation.

## Discussion

SSCs with osteogenic lineage commitment potential play a crucial role in bone formation in adulthood. The underlying cause of systemic bone loss in patients undergoing local radiotherapy remains unclear. Previous studies have confirmed that there is a complex regulatory network between the hematopoietic and skeletal systems, and especially MKs are crucial in bone homeostasis^30,31^. However, the relationship between MKs and SSCs under pathological conditions remains unclear. In this study, we demonstrate that MKs-derived TGFβ1 facilitates zinc ions influx into LepR^+^ SSCs by activating Slc39a14, thereby alleviating ER stress after irradiation. In addition, the increased intracellular zinc levels inhibit PTP1B levels and activate Stat3 signaling, promoting osteogenic lineage commitment of LepR^+^ SSCs.

SSCs not only maintain bone development and homeostasis but also play a key role in the repair of bone injuries^3^. LepR^+^ SSCs are primarily located at the margins of blood vessels in the BM, comprising about 0.3% of the total BM cells^5^. Under normal physiological conditions, most LepR^+^ SSCs remain in a homeostatic quiescent state and are essential for maintaining the homeostasis of the hematopoietic niche^5^. However, LepR^+^ SSCs are rapidly activated in response to damage and are the main source of osteoblasts^5,7^. Our previous research demonstrated that MKs secrete TGFβ1 to induce bone formation. In this study, we used a MK-specific Cre (Pf4-Cre) mouse model. Although a low level of ectopic recombination of Pf4 occasionally occurs, our experimental conditions did not significantly induce or exacerbate this phenomenon. Consequently, this mouse model still demonstrates significant practical value for MK-associated investigations. As a result, targeted deletion of MKs decreased the number of LepR^+^ cells in the BM and suppressed bone formation in the mice. In Pf4-cre; iDTR mice, specific MKs ablation, even with TPO supplementation, failed to restore bone mass. This suggests the osteogenic effect of TPO predominantly mediated by MKs. Although these cell types reside within the BM microenvironment and may exert certain regulatory functions, the primary mechanism by which TPO influences bone homeostasis remains MK dependent. MKs are a critical source of cytokines involved in bone remodeling and in creating the osteogenic microenvironment within the BM niche. Our previous studies have shown that TGFβ1 is highly enriched in MKs, and the ablation of MKs reduces the TGFβ1 concentration in the BM. Moreover, scRNA-seq analysis revealed that the subsets of LepR^+^ SSCs with high expression of TGFβ1 disappeared after irradiation, suggesting that TGFβ1 concentration in the BM is crucial for maintaining bone homeostasis. Indeed, we found that MKs significantly promoted osteogenic lineage commitment of LepR^+^ SSCs. Furthermore, inhibition of the TGFβ1 receptor or conditional deletion of TGFβ1 in MKs significantly weakened the MKs-induced osteogenic differentiation of LepR^+^ SSCs. More importantly, conditional deletion of TGFβ1 in MKs reduced the number of LepR^+^ SSCs in vivo. On the basis of these combined data, we conclude that TGFβ1 plays a major role in MKs-mediated osteogenic lineage commitment of LepR^+^ SSCs. Consistent with our findings, the skeletal TGFβ signaling is suppressed during normal aging of bone^32^. Under various pathological conditions, TGFβ1 may have contradictory effects on bone formation. Decreased serum TGFβ1 shows promising diagnostic potential for osteoporosis^33,34^, while one study revealed an inverse correlation between fracture healing and local TGFβ1 levels in aged mice^35^. Hyperactive TGFβ1 signaling may contribute to skeletal defects in certain disease models^36,37^.

It has been reported that ER stress is associated with the pathological development of osteoporosis^38^. More specifically, the accumulation of unfolded or misfolded proteins can induce ER stress and cause apoptosis in osteoblasts^39^. Under normal circumstances, osteoblasts, chondrocytes and osteoclasts are required to continuously secrete large amounts of extracellular matrix proteins and proteases, placing a heavy protein synthesis burden on these cells, making them susceptible to ER stress. Short-term and mild ER stress can promote osteogenic differentiation of SSCs and MSCs (Mesenchymal stem cells). However, under pathological conditions, prolonged and excessive ER stress result in decreased activity and impaired osteogenic differentiation^40^. In the present study, we confirmed through both in vitro and in vivo experiments that TGFβ1 derived from MKs alleviates ER stress in the BM by increasing intracellular zinc ion concentrations. The zinc transporter Slc39a14 (ZIP14), a zinc importer usually located at the cell surface, is regulated by various cytokines and transcription factor^41^. Slc39a14 controls both physiological and pathological ER stress^42^. In addition, Slc39a14 can upregulate zinc levels and inhibit bone resorption in osteoclasts^43^. Here, we demonstrate that the levels of Slc39a14 and zinc ions are elevated in LepR^+^ SSCs after the addition of MKs from TGFβ1^fl/fl^ mice, but not from TGFβ1^MKΔ/Δ^ mice. Furthermore, we further confirmed that zinc supplementation directly alleviates ER stress and restores osteogenic potential in the context of Slc39a14 deficiency. These findings align with a previous study showing that zinc ions can further reduce ER stress levels^42^. Further verification of the relationship between ER stress inhibition and TGFβ1 signaling was conducted. In experiments where both 4-PBA and a TGFβ inhibitor were used, we observed no significant difference compared to the 4-PBA-only group, indicating that inhibition of ER stress effectively bypasses the negative regulation of the TGFβ1 signaling pathway (Supplementary Fig. 8h,i). This result further supports the hypothesis that ER stress is a downstream event of TGFβ1 signaling. Furthermore, our data reveal that MKs can also secret BMPs (BMP2, BMP4 and BMP6), which may directly participate in bone formation. Thus, MKs are involved in LepR^+^ SSCs osteogenic lineage commitment of LepR^+^ SSCs in the BM a process that is at least partially mediated by TGFβ1.

Protein tyrosine phosphatase-1b (PTP1B), located in the ER, is a member of the protein tyrosine phosphatase family and plays a role in cell signal transduction, as well as the regulation of cell growth and differentiation^44^. Consistent with previous studies, we demonstrated that the increased level of PTP1B in LepR^+^ SSCs after irradiation damage may be related to ER stress. In this work, we found that the expression of PTP1B is higher in LepR^+^ SSCs from the Slc39a14^leprΔ/Δ^ mice compared to those from Slc39a14^fl/fl^ mice after irradiation. Interestingly, we found the specific knockout of Slc39a14 in Lepr-cre cells led to a decrease in bone mass, indicating that Slc39a14 plays a crucial role in bone mass maintenance. In line with prior research, our data suggest that intracellular zinc ions are crucial for downregulating PTP1B levels in LepR^+^ SSCs^45^. Subsequently, systemic delivery of TPO was used to treat the irradiated Slc39a14^leprΔ/Δ^ mice to maintain the amount of MKs. However, no improvement in radiation-induced bone loss was observed. In addition, we demonstrate that PTP1B negatively regulates stat3 phosphorylation, which impacts osteogenic differentiation in LepR^+^ SSCs. Taken together, the reduction in LepR^+^ SSCs may offer a plausible explanation for radiation-induced bone loss, which can be alleviated by TGFβ1 derived from MKs.

In conclusion, our findings demonstrate that MKs play a crucial role in bone formation by inhibiting ER stress in LepR^+^ SSCs, at least in part through secreting TGFβ1. In addition, this study suggests that the crosstalk between MKs and LepR^+^ SSCs offers a potential therapeutic target for bone loss induced by irradiation. Therefore, targeting the regulation of MKs may provide valuable insights for the treatment of radiation-induced bone loss.

## Supplementary information

Supplementary Information

## Data Availability

The data of RNA sequencing supporting the findings of this study are deposited in the National Center for Biotechnology Information (NCBI) database (PRJNA561251). Any additional information required to reanalyze the data reported in this Article is available from the lead contact upon request.
