# Supplementary material for: Megakaryocytic TGFβ1 orchestrates osteogenesis of LepR+ SSCs to alleviate radiation-induced bone loss
Source: Exp Mol Med. 2026 Jan 14;58(1):161–77. doi: 10.1038/s12276-025-01612-z (PMC12868635; doi:10.1038/s12276-025-01612-z)
Supplement: Supplementary file 1 — Supplementary Information [file 12276_2025_1612_MOESM1_ESM.docx]

**Megakaryocytic TGF-β1 orchestrates osteogenesis of LepR^+^ SSCs to alleviate radiation-induced bone loss**

**Supplemental information**

**Materials and Methods**

**Flow cytometry sorting**

For primary LepR^+^ SSCs sorting, BM plugs from femurs and tibiae were flushed and dissociated. For osteoblast sorting, femurs and tibiae of Col2.3-tdTomato mice were crushed by mortar and pestle, followed by enzymatic dissociation and antibody staining to sort for DAPI^-^CD45^-^Ter119^-^CD31^-^Col2.3-tdTomato^+^ cells. For bone marrow endothelial cell sorting, mice received a tail vein injection of Alexa Fluor 647 anti-CD144 antibody (10 µg/mouse, Biolegend, clone: BV13) for 10 minutes for in vivo labeling. Following crushing, enzymatic dissociation, and DAPI staining of femurs and tibiae, DAPI^-^CD144^+^ cells were subsequently sorted. For hematopoietic analysis, whole bone marrow (BM) cells were flushed and dissociated into single-cell suspensions in staining buffer (Ca2^+^/Mg2^+^-free HBSS with 2% FBS). Cells were subsequently stained with fluorescently conjugated antibodies to identify specific lineages: myeloid (CD11b^+^Gr-1^+^), erythroid (Ter119^+^CD71^+^), B cells (B220^+^), and T cells (CD3^+^).

**Lentivirus transfection**

The level of PTP1B was suppressed or overexpressed by lentivirus transfection (Tsingke Biotechnology Co., Ltd., Beijing, China). Lentiviral transfection was conducted according to previously described methods and the manufacturer's protocol. Primary skeletal stem cells were infected with lentivirus at a multiplicity of infection (MOI) of 40 in the presence of 8 μg/mL polybrene. After 24 h, culture medium was replaced, and cells were further cultured for 24 h before puromycin selection (5 μg/mL). Transduction efficiency was confirmed by GFP fluorescence, and PTP1B expression was validated by western blotting. Then, the medium was changed tonormal complete medium supplemented with puromycin.


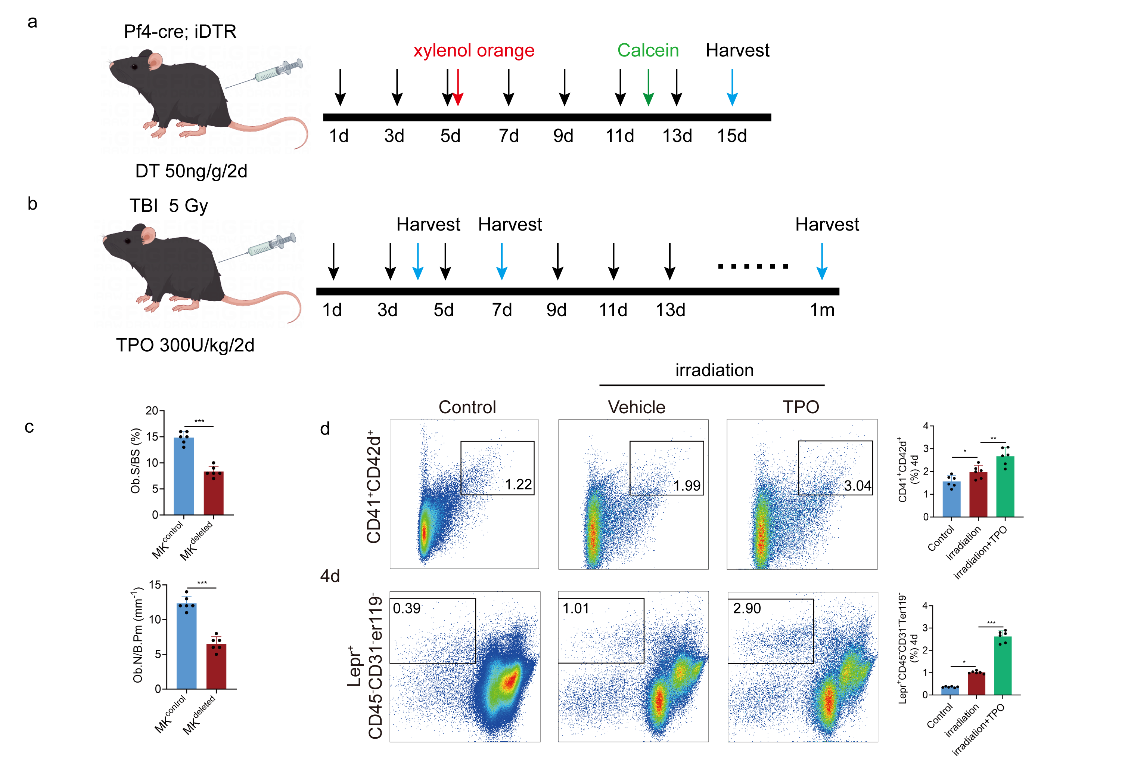


**Supplementary Fig. 1. (a)** Scheme for DT administration to Pf4-cre^+^;iDTR and Pf4-cre^-^;iDTR mice. **(b)** Scheme for TPO administration to the irradiated mice. **(c)** Bone histomorphometry parameters (Ob.S/BS, Ob.N/B.Pm) at the distal femur metaphysis from MK^deleted^ mice and their littermate controls (n=6 mice per group). **(d)** Representative flow cytometry plots and quantification of percent MKs (CD41^+^CD42d^+^) and LepR^+^ SSCs (Lepr^+^CD45^-^CD31^-^Ter119^-^) 4 days after irradiation (n=6 mice per group). Data on graphs are shown as mean ± SD. One-way ANOVA was used to analyze the data in (d). *P < 0.05, **P < 0.01, ***P < 0.001. For all panels in this figure, data are representative of three independent experiments. WBM: whole bone marrow.


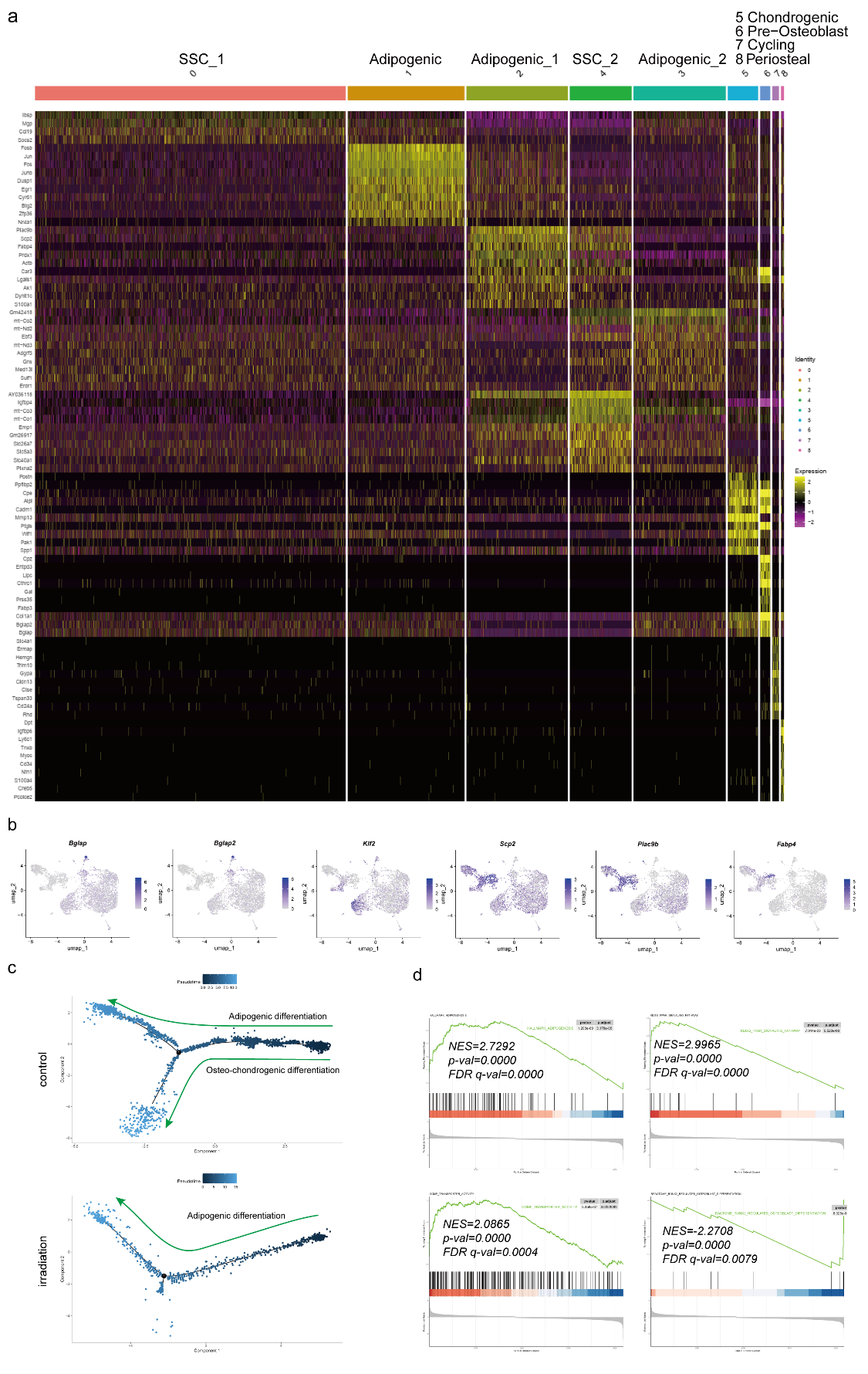


**Supplementary Fig. 2. (a)** Heatmap illustrating the relative expression levels (row-wise Z score) of the 84 most significant markers for each cluster (rows) across cells in the 9 clusters (columns). **(b)** Dot plots showing the gene expression patterns of selected genes in each cluster. **(c)** Pseudotime analysis within Lepr-cre-traced osteo-chondrogenic and adipogenic lineage cells under control and irradiation conditions. **(d)** Gene set enrichment analysis (GSEA) plots showing upregulation of adipogenesis, PPARγ signaling pathway, transporter activity and osteoblast differentiation.


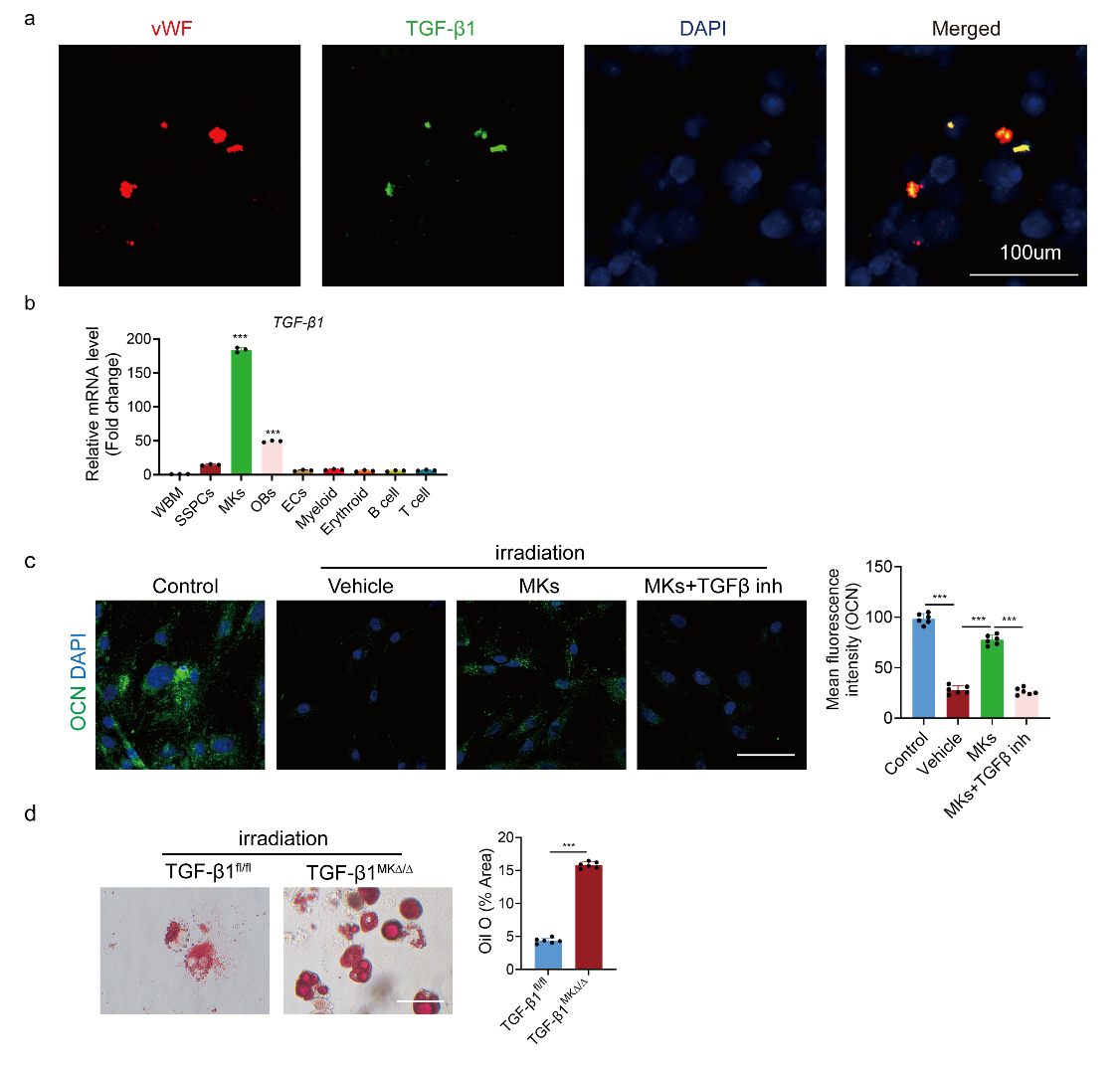


**Supplementary Fig. 3. (a)** Representative image of a bone marrow section from a C57BL/6J mouse showing TGF-β1 staining in MKs. **(b)** qPCR analyses of TGF-β1 expression in 8-week-old long bones, WBM (Whole BM cells); SSCs (CD45^-^Ter119^-^CD31^-^Lepr-tdTomato^+^; MKs (CD41^+^CD42d^+^); osteoblasts (OBs, ALP^+^); endothelial cells (ECs, CD144^+^); myeloid (CD11b^+^Gr-1^+^); erythroid (Ter119^+^CD71^+^); B cells (B220^+^); T cells (CD3) (n=3 mice from three independent experiments). **(c)** Representative immunostaining images of OCN (green) in LepR^+^ SSCs, with or without MKs, from the BM of TGF-β1^MK∆/∆^ and TGF-β1^fl/fl^ mice (n=6 per group). **(d)** LepR^+^ SSCs were induced in adipogenic differentiation medium with or without MKs from the BM of TGF-β1^MK∆/∆^ and TGF-β1^fl/fl^ mice after 21 days. Representative Oil O staining images (left) and quantification of area was calculated (right) (n=6 per group). Scale bar, 100 µm. Data on graphs are shown as mean ± SD. One-way ANOVA was used to analyze the data in (b, c) and an unpaired two-tailed t-test was used to analyze the data in (d). *P < 0.05, **P < 0.01, ***P < 0.001. For all panels in this figure, data are representative of three independent experiments.


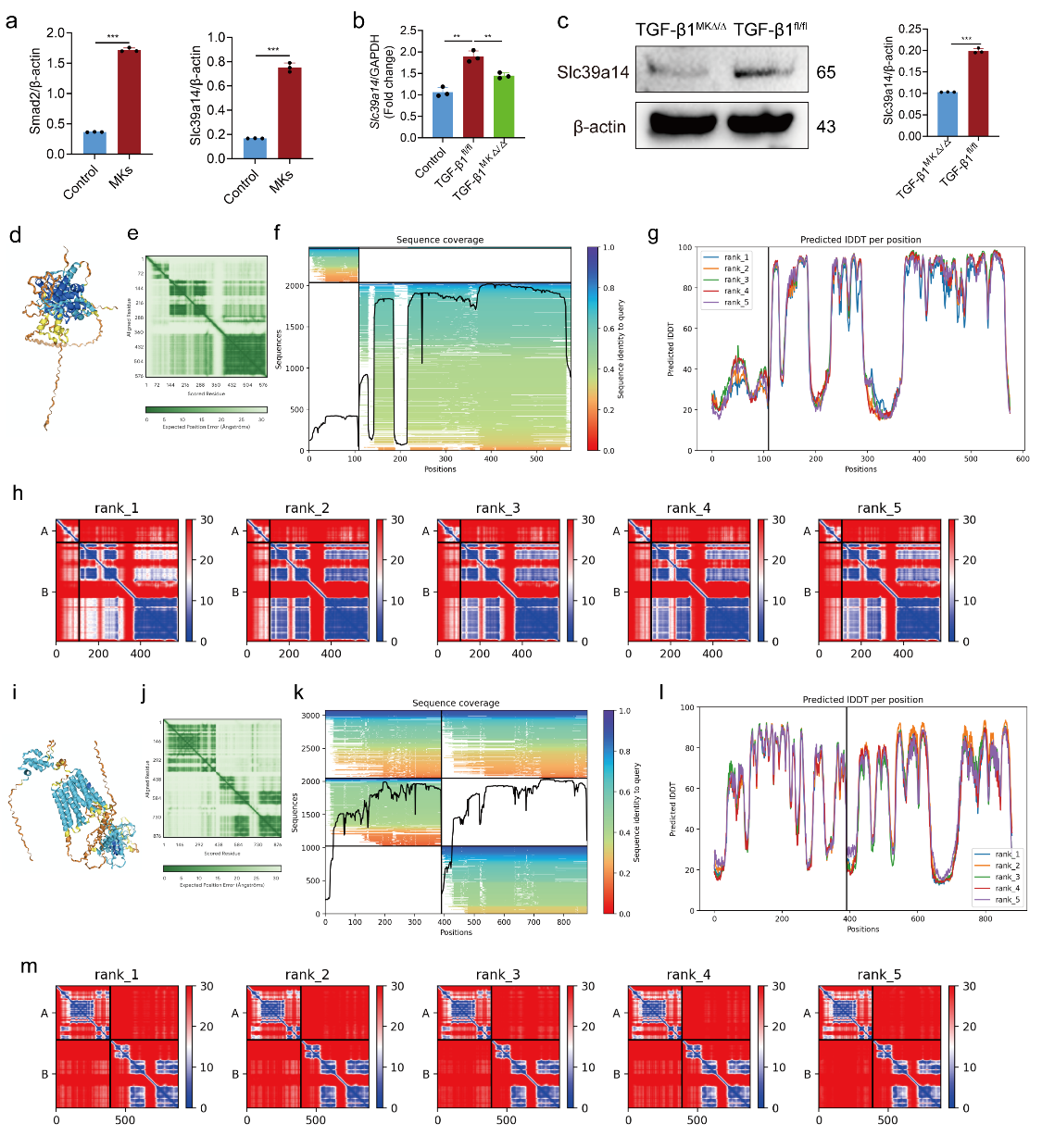


**Supplementary Fig. 4. (a)** Western blotting analysis of smad2 and slc39a14 expression in LepR^+^ SSCs, with or without MKs, from WT mice. (n=3 per group). **(b)** qPCR analysis of *Slc39a14* expression in LepR^+^ SSCs with MKs from the BM of TGF-β1^MK∆/∆^ and TGF-β1^fl/fl^ mice (n=3 per group). **(c)** Western blotting analysis of Slc39a14 expression in LepR^+^ SSCs with MKs from the BM of TGF-β1^MK∆/∆^ and TGF-β1^fl/fl^ mice (n=3 per group). **(d)** Schematic representation of the neural network model of Smad2 binding to Slc39a14 protein predicted by Alphafold3. **(e)** Plot of the predicted aligned error of the complex, predicted by Alphafold3. **(f)** Sequence comparison results for Smad2 and Slc39a14. **(g)** Predicted IDDT per position of five trained neural networks for Smad2 and Slc39a14. **(h)** Predicted aligned errors of five trained neural networks for Smad2 and Slc39a14. **(i)** Schematic representation of the neural network model of TGF-β1 binding to Slc39a14 protein, predicted by Alphafold3. **(j)** Plot of the predicted aligned error of the complex, predicted by Alphafold3. **(k)** Sequence comparison results for TGF-β1 and Slc39a14. **(l)** Predicted IDDT per position of five trained neural networks for TGF-β1 and Slc39a14. **(m)** Predicted aligned errors of five trained neural networks for TGF-β1 and Slc39a14. Data on graphs are shown as mean ± SD. One-way ANOVA was used to analyze the data in (b) and an unpaired two-tailed t-test was used to analyze the data in (a, c). *P < 0.05, **P < 0.01, ***P < 0.001. For all panels in this figure, data are representative of three independent experiments.


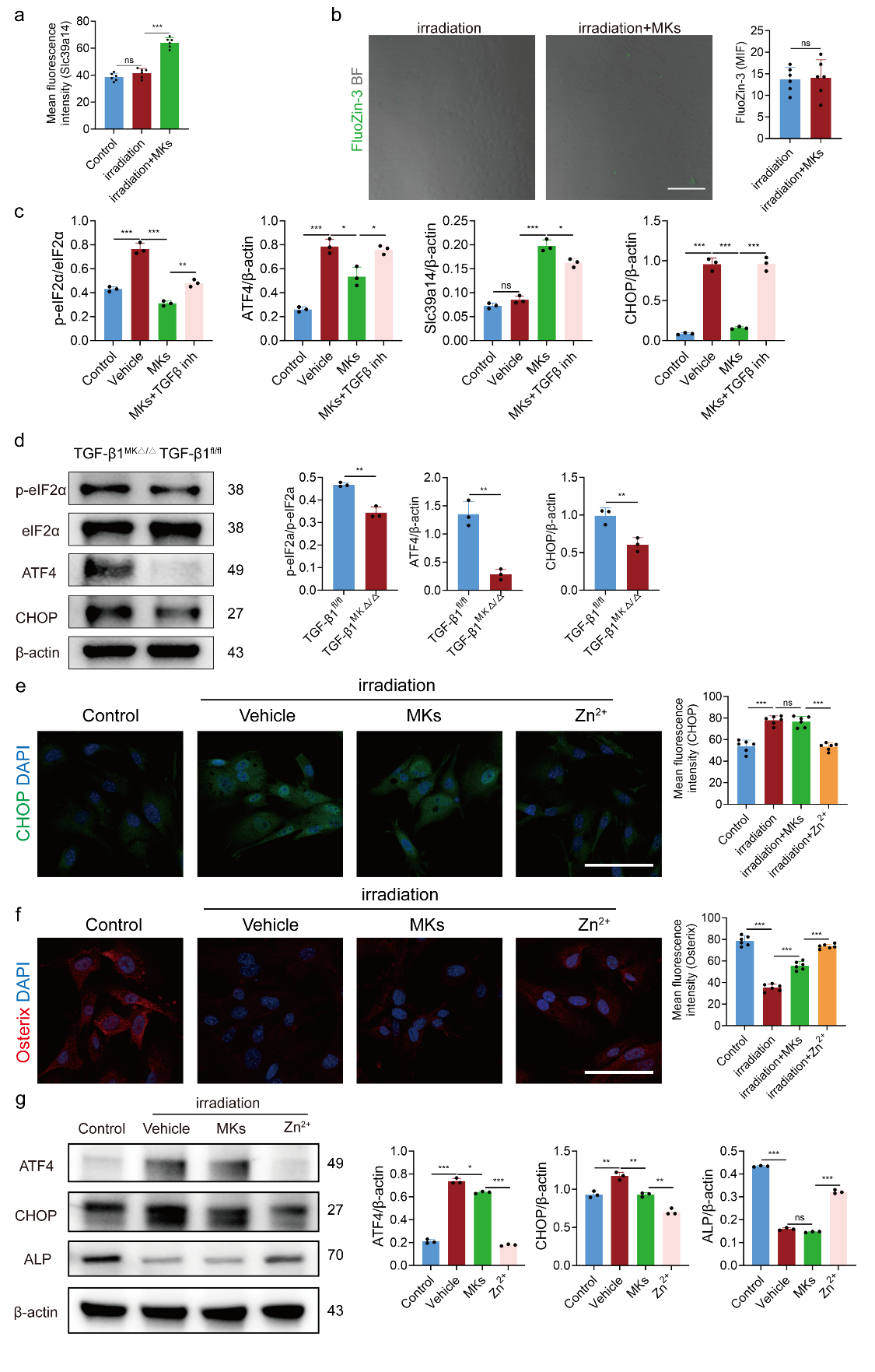


**Supplementary Fig. 5. (a)** Quantitative analysis of LepR^+^ SSCs with Slc39a14, with or without MKs, after irradiation (n=6 per group). **(b)** Representative fluozin-3 images of LepR^+^ SSCs with Slc39a14 deletion, with or without MKs, after irradiation (n=6 per group). **(c)** Western blotting analysis of the expression of Slc39a14, PTP1B, p-eIF2α, ATF4 and CHOP in LepR^+^ SSCs after coculture with MKs (n = 3 per group). **(d)** Western blotting analysis of the expression of p-eIF2α, ATF4 and CHOP in LepR^+^ SSCs after coculture with MKs from the BM of TGF-β1^MK∆/∆^ and TGF-β1^fl/fl^ mice (n = 3 per group). **(e)** Representative immunostaining images of CHOP (green) in Slc39a14 KO LepR^+^ SSCs, with MKs or zinc acetate (10uM), after irradiation (n=6 per group). Scale bar, 100 µm. **(f)** Representative immunostaining images of OCN (red) in Slc39a14 KO LepR^+^ SSCs, with MKs or zinc acetate (10uM), after irradiation (n=6 per group). Scale bar, 100 µm. **(g)** Western blotting analysis of the expression of ATF4, CHOP and ALP in Slc39a14 KO LepR^+^ SSCs after coculture with MKs or zinc acetate (10uM), after irradiation (n = 3 per group). Data on graphs are shown as mean ± SD. One-way ANOVA was used to analyze the data in (a, c, e-g) and an unpaired two-tailed t-test was used to analyze the data in (b, d). *P < 0.05, **P < 0.01, ***P < 0.001. For all panels in this figure, data are representative of three independent experiments.


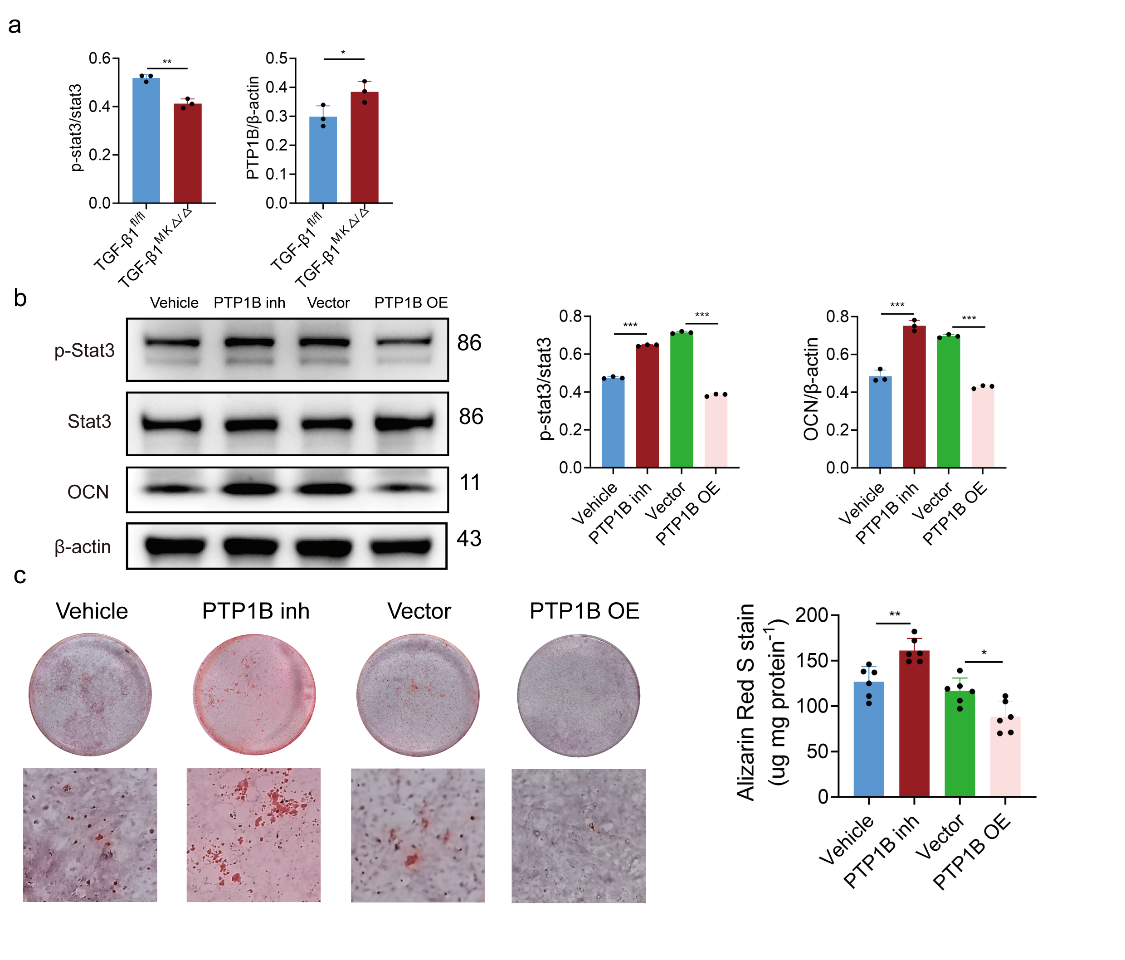


**Supplementary Fig. 6. (a)** Western blotting analysis of the expression of PTP1B and p-Stat3 in LepR^+^ SSCs after coculture with MKs from the BM of TGF-β1^MK∆/∆^ and TGF-β1^fl/fl^ mice (n = 3 per group). **(b)** Western blotting analysis of the expression of p-Stat3 and OCN in LepR^+^ SSCs with PTP1B inhibitor (PTP1B-IN-1) or overexpression of PTP1B by lentivirus (n = 3 per group). **(c)** LepR^+^ SSCs were induced in osteogenic differentiation medium with PTP1B inhibitor (PTP1B-IN-1) or overexpression of PTP1B by lentivirus after 21 days. Representative Alizarin red staining images (left) and quantification of matrix mineralization was calculated (right) (n=6 per group). Data on graphs are shown as mean ± SD. One-way ANOVA was used to analyze the data in (b, c). An unpaired two-tailed t-test was used to analyze the data in (a). *P < 0.05, **P < 0.01, ***P < 0.001. For all panels in this figure, data are representative of three independent experiments.


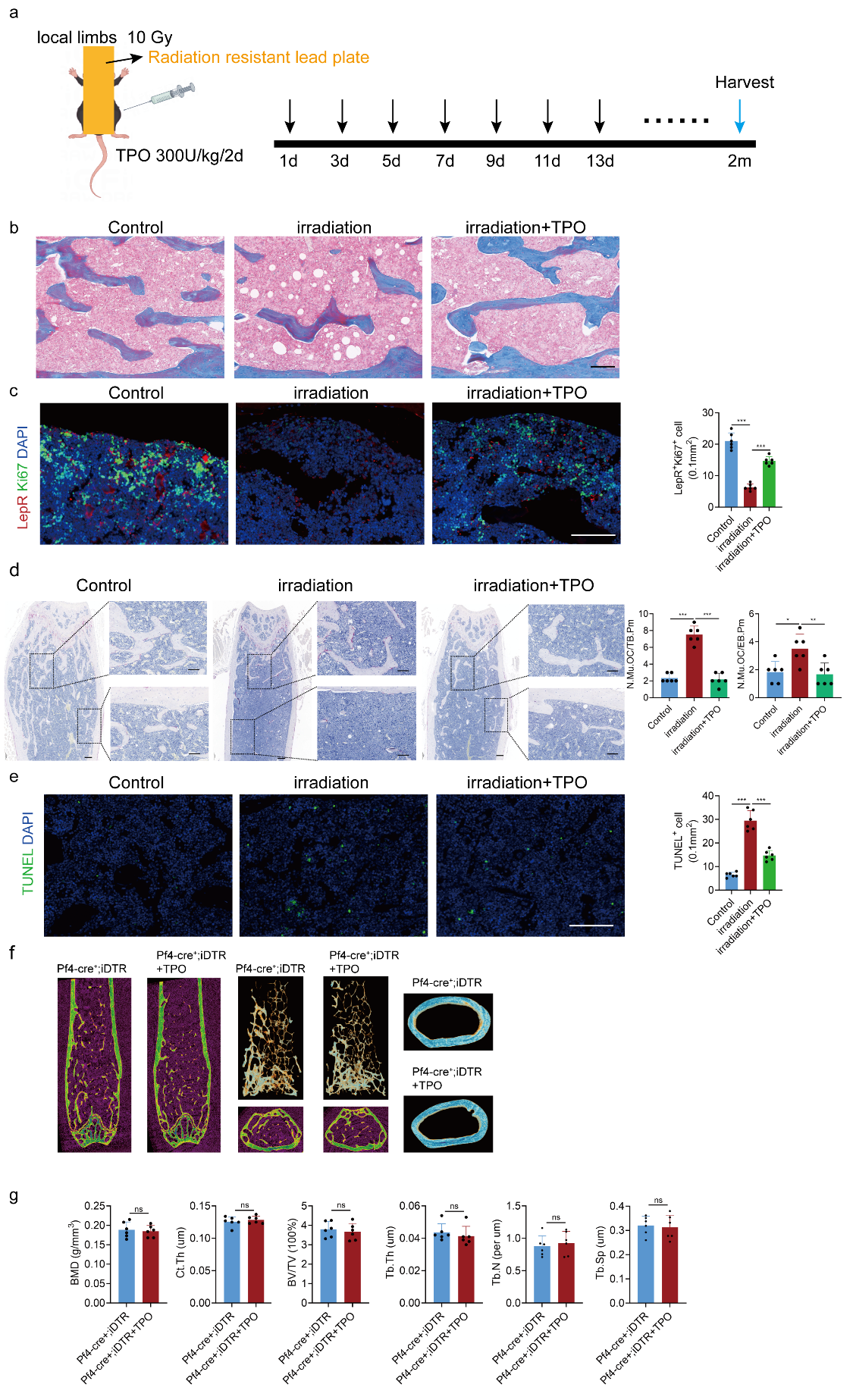


**Supplementary Fig. 7 (a)** Scheme for TPO administration to the irradiated mice. **(b)** Masson’s trichrome staining showing the osteogenic niche in control or irradiated mice 8 weeks after injection with TPO (n=6 mice per group). Scale bar, 100 µm. **(c)** Colocalization of LepR (red) with Ki67 (green) in the bone marrow (BM) of irradiation mice (n=6 mice per group). Scale bar, 100 µm. **(d)** TRAP staining of distal femur sections from mice injected with TPO or vehicle after irradiation for 8 weeks, along with quantification of multinucleated osteoclasts (n=6 mice per group). **(e)** Representative immunostaining images of TUNEL (green) in the bone marrow of mice injected with TPO or vehicle after irradiation. The quantification of TUNEL-positive cells is shown in the right panel (n=6 mice per group). **(f)** Representative Micro-CT images of longitudinal section femurs, cross-sectional view of the distal femurs and reconstructed trabecular structure of the region of interest (ROI) from Pf4-cre^+^; iDTR mice injected with TPO or vehicle (n=6 mice per group). **(g)** Quantitative Micro-CT analysis of the trabecular bone fraction (BV/TV, Tb.N, Tb.Th, Tb.Sp, BMD and Ct.Th) in Pf4-cre^+^; iDTR mice injected with TPO or vehicle (n=6 mice per group). Scale bar, 100 µm. Data on graphs are shown as mean ± SD. One-way ANOVA was used to analyze the data in (c, d, e), an unpaired two-tailed t-test was used to analyze the data in (g). *P < 0.05, **P < 0.01, ***P < 0.001. For all panels in this figure, data are representative of three independent experiments.


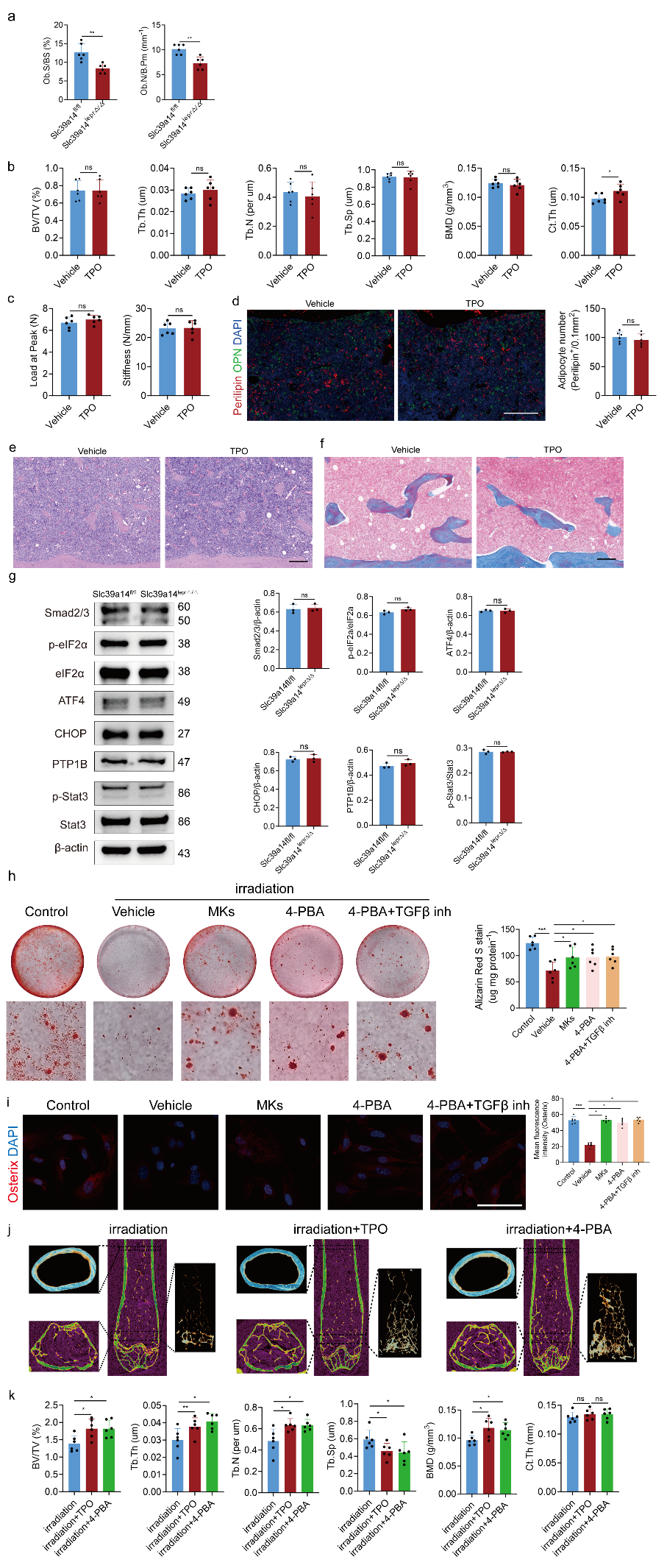


**Supplementary Fig. 8. (a)** Bone histomorphometry parameters, including Ob.S/BS (the percentage of trabecular bone surface covered by osteoblasts) and Ob.N/B.Pm (osteoblast number/bone perimeter), at the distal femur metaphysis from Slc39a14^lepr∆/∆^ mice and their littermate controls (Slc39a14^fl/fl^ mice) (n=6 mice per group). **(b)** Quantitative Micro-CT analysis of the trabecular bone fraction (BV/TV, Tb.N, Tb.Th, Tb.Sp, BMD and Ct.Th) in Slc39a14^lepr∆/∆^ mice injected with TPO or vehicle after irradiation (n=6 mice per group). **(c)** Quantitative biomechanical analysis of femora (Peak load and stiffness) from Slc39a14^lepr∆/∆^ mice injected with TPO or vehicle after irradiation (n=6 mice per group). **(d)** Representative immunostaining images of Perilipin (red) and osteopontin (OPN, green) in the bone marrow (BM) of Slc39a14^lepr∆/∆^ mice injected with TPO or vehicle after irradiation (n=6 mice per group). Scale bar, 100 µm. **(e)** HE staining showing the osteogenic niche of the Slc39a14^lepr∆/∆^ mice injected with TPO or vehicle after irradiation (n=6 mice per group). Scale bar, 100 µm. **(f)** Masson staining showing the osteogenic niche of the Slc39a14^lepr∆/∆^ mice injected with TPO or vehicle after irradiation (n=6 mice per group). **(g)** Western blotting analysis of the expression of slc39a14, PTP1B, p-eIF2α, ATF4, CHOP, PTP1B and p-stat3 in LepR^+^ SSCs after co-culture with MKs from the BM of Slc39a14^lepr∆/∆^ and their littermate controls (Slc39a14^fl/fl^ mice) (n = 3 per group). **(h)** LepR^+^ SSCs were induced in osteogenic differentiation medium with ER stress inhibitor 4-phenylbutyric acid (4-PBA) or pretreated TGF-β type I receptor inhibitor SB431542 after 21 days. Representative Alizarin red staining images (left) and quantification of matrix mineralization was calculated (right) (n=6 per group). **(i)** Representative immunostaining images of osterix (red) in LepR^+^ SSCs, with ER stress inhibitor 4-phenylbutyric acid (4-PBA) or pretreated TGF-β type I receptor inhibitor SB431542 (n=6 per group). Scale bar, 100 µm. **(j)** Representative Micro-CT images of longitudinal section femurs, cross-sectional view of the distal femurs and reconstructed trabecular structure of the region of interest (ROI) from mice injected with TPO or ER stress inhibitor 4-phenylbutyric acid (4-PBA) (n=6 mice per group). **(k)** Quantitative Micro-CT analysis of the trabecular bone fraction (BV/TV, Tb.N, Tb.Th, Tb.Sp, BMD and Ct.Th) in mice injected with TPO or ER stress inhibitor 4-phenylbutyric acid (4-PBA) (n=6 mice per group). Data on graphs are shown as mean ± SD. An unpaired two-tailed t-test was used to analyze the data in (a-d, g). One-way ANOVA was used to analyze the data in (h, i, k). *P < 0.05, **P < 0.01, ***P < 0.001. For all panels in this figure, data are representative of three independent experiments.

**Supplementary Table 1**

**Primer sequences**

| **Gene** |  | **Sequence (5' -> 3')** |
| --- | --- | --- |
| Osterix | Forward Primer | GGAAAGGAGGCACAAAGAAGC |
|  | Reverse Primer | CCCCTTAGGCACTAGGAGC |
| Runx2 | Forward Primer | GACTGTGGTTACCGTCATGGC |
|  | Reverse Primer | ACTTGGTTTTTCATAACAGCGGA |
| Slc39a14 | Forward Primer | GAGTGGGCCGGGATAATGTTT |
|  | Reverse Primer | GAGATCGCTCGCTCAAGTTGT |
| Smad2 | Forward Primer | AAGCCATCACCACTCAGAATTG |
|  | Reverse Primer | CACTGATCTACCGTATTTGCTGT |
| Adipoq | Forward Primer | TGTTCCTCTTAATCCTGCCCA |
|  | Reverse Primer | CCAACCTGCACAAGTTCCCTT |
| PPARγ | Forward Primer | GCGAGGGCGATCTTGACA |
|  | Reverse Primer | CTTGCACGGCTTCTACGG |
| GAPDH | Forward Primer | CCTCGTCCCGTAGACAAAATG |
|  | Reverse Primer | TCTCCACTTTGCCACTGCAA |
